# Effect of smoking on the diagnostic results and complication rates of percutaneous transthoracic needle biopsy

**DOI:** 10.1007/s00330-024-10705-8

**Published:** 2024-03-25

**Authors:** Woo Hyeon Lim, Jong Hyuk Lee, Hyungin Park, Chang Min Park, Soon Ho Yoon

**Affiliations:** 1 Department of Radiology, Seoul National University Hospital, 101 Daehak-ro, Jongno-gu, Seoul, 03080 Korea; 2 Department of Radiology, Seoul National University College of Medicine, 101 Daehak-ro, Jongno-gu, Seoul, 03080 Korea

**Keywords:** Image-guided biopsy, Lung neoplasms, Diagnosis, Pneumothorax, Hemoptysis

## Abstract

**Objective:**

To investigate the association of smoking with the outcomes of percutaneous transthoracic needle biopsy (PTNB).

**Methods:**

In total, 4668 PTNBs for pulmonary lesions were retrospectively identified. The associations of smoking status (never, former, current smokers) and smoking intensity (≤ 20, 21–40, > 40 pack-years) with diagnostic results (malignancy, non-diagnostic pathologies, and false-negative results in non-diagnostic pathologies) and complications (pneumothorax and hemoptysis) were assessed using multivariable logistic regression analysis.

**Results:**

Among the 4668 PTNBs (median age of the patients, 66 years [interquartile range, 58–74]; 2715 men), malignancies, non-diagnostic pathologies, and specific benign pathologies were identified in 3054 (65.4%), 1282 (27.5%), and 332 PTNBs (7.1%), respectively. False-negative results for malignancy occurred in 20.5% (236/1153) of non-diagnostic pathologies with decidable reference standards. Current smoking was associated with malignancy (adjusted odds ratio [OR], 1.31; 95% confidence interval [CI]: 1.02–1.69; *p *= 0.03) and false-negative results (OR, 2.64; 95% CI: 1.32–5.28; *p *= 0.006), while heavy smoking (> 40 pack-years) was associated with non-diagnostic pathologies (OR, 1.69; 95% CI: 1.19–2.40; *p *= 0.003) and false-negative results (OR, 2.12; 95% CI: 1.17–3.92; *p *= 0.02). Pneumothorax and hemoptysis occurred in 21.8% (1018/4668) and 10.6% (495/4668) of PTNBs, respectively. Heavy smoking was associated with pneumothorax (OR, 1.33; 95% CI: 1.01–1.74; *p *= 0.04), while heavy smoking (OR, 0.64; 95% CI: 0.40–0.99; *p *= 0.048) and current smoking (OR, 0.64; 95% CI: 0.42–0.96; *p *= 0.04) were inversely associated with hemoptysis.

**Conclusion:**

Smoking history was associated with the outcomes of PTNBs. Current and heavy smoking increased false-negative results and changed the complication rates of PTNBs.

**Clinical relevance statement:**

Smoking status and intensity were independently associated with the outcomes of PTNBs. Non-diagnostic pathologies should be interpreted cautiously in current or heavy smokers. A patient’s smoking history should be ascertained before PTNB to predict and manage complications.

**Key Points:**

*• Smoking status and intensity might independently contribute to the diagnostic results and complications of PTNBs.*

*• Current and heavy smoking (> 40 pack-years) were independently associated with the outcomes of PTNBs.*

*• Operators need to recognize the association between smoking history and the outcomes of PTNBs.*

**Supplementary Information:**

The online version contains supplementary material available at 10.1007/s00330-024-10705-8.

## Introduction

Cigarette smoking is a well-established risk factor for major pulmonary diseases, including lung cancer [1, 2], chronic obstructive pulmonary disease [3], and idiopathic pulmonary fibrosis [4]. Smoking induces chronic inflammation, which can directly and indirectly affect the airway and lung parenchyma [5], leading to pathologic changes [6–8]. Furthermore, smoking can also impair pulmonary endothelial function [9].

Percutaneous transthoracic needle biopsy (PTNB) is a less-invasive diagnostic procedure used to obtain tissue specimens. This procedure is relatively safe, with a 21.7% rate of pneumothorax and a 5.2% rate of hemoptysis, and highly accurate (diagnostic accuracy, 91.1%) [10, 11]. Therefore, PTNB is typically performed to diagnose indeterminate pulmonary lesions, especially for peripheral lesions suspicious of lung cancer.

After the implementation of lung cancer screening programs, the role of PTNB in the diagnosis of pulmonary lesions has become more important, given the increasing incidence of peripheral lung cancer [12]. As the eligibility criteria for lung cancer screening include specific requirements for smoking history [13–15], it is necessary to understand the impact of smoking on PTNB. Smoking might be an important factor associated with the complication rates and pathologic diagnosis of PTNB procedures, given the physiologic and pathologic changes induced by smoking [5–9]. For example, impaired pulmonary function and structural changes in the lungs in smokers could contribute to complications [5–9]. In addition, smoking-induced chronic inflammation might affect the diagnosis of pulmonary lesions using PTNB specimens [6–8].

However, the effect of smoking on the outcomes of PTNB has not been investigated extensively. Although it has been observed that malignancy rates are higher in patients with greater smoking intensity [16], prior studies on the outcomes of PTNBs have only considered smoking status (ever-smoker vs. never smoker) [10, 11]. To accurately determine impact of smoking on the outcomes of PTNB, ever-smokers should be stratified further into former and current smokers since current smoking status is associated with inflammation [17], and smoking intensity also needs to be considered independently.

In this clinical context, the present study sought to investigate whether smoking status and intensity affected the diagnostic results and complication rates of PTNB for pulmonary lesions.

## Materials and methods

This retrospective study was approved by the Institutional Review Board of Seoul National University Hospital, with a waiver of informed consent. Data from some patients were previously reported [18–21], but none of those studies had an exact overlap with the study patients or focused on the association between smoking and PTNB.

### Patients

From January 2013 to June 2019, all PTNB procedures from our institutional registry were identified. This registry consisted of consecutive PTNBs performed for intrathoracic lesions requiring a pathologic diagnosis (based on the shared decisions made by clinicians and thoracic radiologists) at a tertiary referral hospital. Common indications for PTNBs included (1) growing pulmonary lesions, (2) persistent pulmonary lesions or multiple nodular pulmonary lesions of unknown etiology, (3) non-pulmonary lesions (i.e., mediastinal or pleural lesions) of unknown etiology [22]. The inclusion criteria of this study were as follows: (1) patients with available information on their smoking history in the electronic medical records, and (2) patients who underwent their first biopsy for a pulmonary lesion during the study period.

**Fig. 1:**
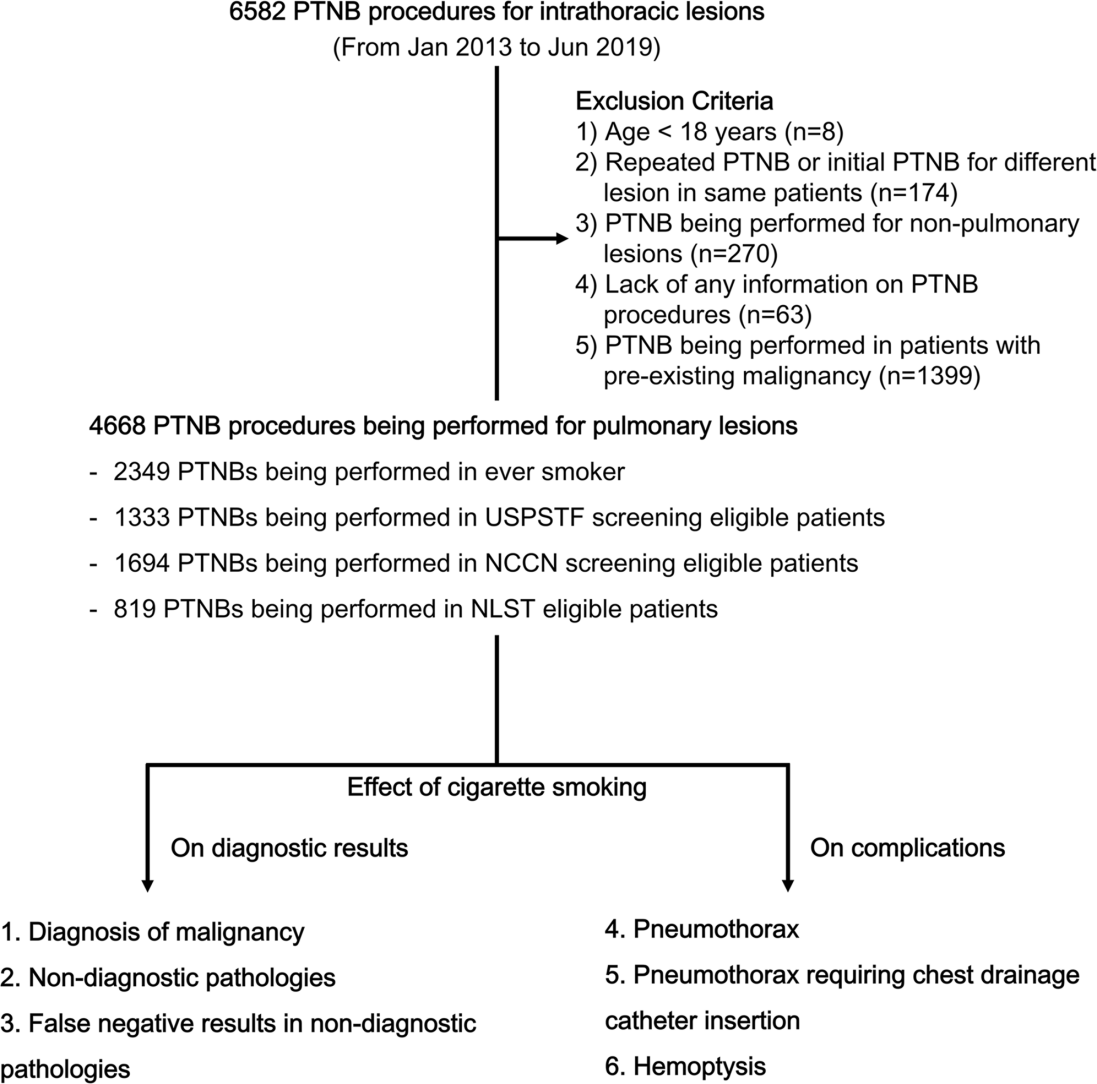
Study flow diagram

The exclusion criteria were as follows: (1) age < 18 years, (2) repeated biopsy or initial biopsy for a different lesion in the same patient during the study period, (3) biopsy performed for non-pulmonary lesions, (4) lack of any information on the biopsy procedures utilized for the analysis (i.e., biopsy needle size, the number of pleural passages, the number of tissue samples, or needle indwelling time), (5) biopsy performed in patients with a pre-existing malignancy (either intrathoracic or extrathoracic) (Fig. 1).

### Biopsy procedures

All the PTNB procedures were performed either by board-certified thoracic radiologists under the supervision of attending thoracic radiologists or attending thoracic radiologists alone. The majority of the PTNB procedures were performed by thoracic radiologists taking part in fellowship training (Method S1). Two cone-beam CT machines were used during the study period (Axiom Artis dTA/VB30, Siemens; Allura Xper FD20, Philips Healthcare).

The target lesion, PTNB technique (coaxial technique vs. fine needle aspiration only), and needle size were chosen by the operators [19–21, 23]. On-site pathologists did not attend the PTNB procedures, and the operators chose the number of tissue samples based on the adequacy of the PTNB specimen (Method S1).

### Data collection

Smoking history (smoking status: never smoker, former smoker, or current smoker; and pack-years) and demographic information (age and sex) were obtained from electronic medical records. Smoking history was collected by self-reporting. Individuals who smoked 100 cigarettes in their lifetime were considered ever-smokers [24]. Former smokers were defined as patients who stopped smoking more than 30 days before the PTNB, and patients who continued smoking were considered current smokers [24]. Patients were stratified according to their smoking intensity: (1) never-smokers or light smokers (≤ 20 pack-years), (2) moderate smokers (> 20 and ≤ 40 pack-years), and (3) heavy smokers (> 40 pack-years) [25]. Data on target lesions, PTNB procedures, and PTNB-associated outcomes (complications, pathologic results) were collected either by the first author (W.H.L.) or a study coordinator (Method S2).

### Diagnostic results

If a primary malignancy was diagnosed based on the PTNB specimen, the PTNB procedure was classified as a true-positive. Conversely, if a specific benign disease (such as tuberculosis, chondroid hamartoma, or sclerosing pneumocytoma) was diagnosed, the PTNB procedure was considered to be a true negative.

Non-diagnostic pathologies were defined when the PTNB specimens showed non-specific benign findings (such as chronic inflammation, non-neoplastic lung parenchyma, fibrosis, or necrosis), atypical cells only, or insufficient specimens to reach a specific diagnosis [10, 26].

Non-diagnostic PTNB procedures were considered false-negatives if malignancies were identified on additional biopsies (repeated PTNB, transbronchial biopsy, or surgical biopsy). If lesions significantly decreased in size (more than 20% in diameter) on follow-up (either by CT or plain chest radiographs) or were stable for 2 years without treatment, PTNB procedures were defined as true-negatives [10]. In cases with follow-up shorter than 2 years, non-diagnostic PTNB procedures were classified as undecidable [10].

### Statistical analysis

After testing normality with the Shapiro-Wilk test, continuous variables showing a parametric distribution were presented as the mean and standard deviation, and those with a non-parametric distribution were presented as the median and interquartile range. Continuous variables were compared using a one-way analysis of variance, the Kruskal-Wallis test, as appropriate, while frequencies were compared using the chi-square test. The Bonferroni correction was applied for multiple pairwise comparisons. The 95% confidence interval (CI) for rates was calculated using the Clopper-Pearson method [27].

The effect of smoking on the outcomes of PTNB procedures was evaluated with adjustments for covariates based on previous studies [10, 11, 16, 26]. The association between smoking and the outcomes of PTNB procedures was also assessed in PTNBs performed using the core needle biopsy. As sensitivity analyses, multivariable logistic regression with backward elimination was performed without univariate screening (Method S3) [28].

The associations between eligibility according to the US Preventive Services Task Force (USPSTF) [13], National Comprehensive Cancer Network (NCCN) [14], or National Lung Screening Trial (NLST) [15] criteria, and the outcomes of PTNB were also explored using multivariable logistic regression analysis with backward elimination (Method S3).

This study considered the operators’ proficiency in PTNB procedures and the difficulty of PTNB procedures. The operators’ experience, the presence of emphysema along the needle tract, the presence of the open bronchus sign in the target lesion, and needle indwelling time were adjusted for the analyses (Method S3) [19, 29, 30].

All statistical analyses were performed with R (version 4.1.2), and a *p* value of 0.05 was set as the threshold for statistical significance. A variance inflation factor greater than 10 was regarded as indicating the presence of multi-collinearity.

## Results

Among 6582 PTNB procedures, 4668 performed for pulmonary lesions (size range, 0.5–19.0 cm) were finally included (patients’ median age, 66 years [interquartile range: 58, 74]; 2715 men [58.2%]; 2349 ever-smokers [50.3%]). A study flow diagram is displayed in Fig. 1.

Table 1 summarizes the demographics, lesion characteristics, and procedural factors according to smoking intensity. In brief, age, sex, smoking status, pack-years, lesion size, lesion type, location of lesion, the proportion of lesions with the open bronchus sign, and the rates of emphysema and emphysema along the needle tract detected on CT significantly differed according to smoking intensity (*p* < 0.05). The operators’ experience, procedure type, and needle indwelling time were not significantly different among the groups (*p* > 0.05).

**Table 1:** Demographics, lesion characteristics, and procedural factors according to smoking intensity

	Never or light smoker (*n* = 3037)^a^	Moderate smoker (*n* = 921)^a^	Heavy smoker (*n* = 710)^a^	*p* value
Age^b^	65 [56, 73]	67 [60, 74]	71 [66, 76]	< 0.001
Sex				< 0.001
Men (*n* = 2715; 58.2%)	1122 (36.9%)	888 (96.4%)	705 (99.3%)	
Women (*n* = 1953; 41.8%)	1915 (63.1%)	33 (3.6%)	5 (0.7%)	
Smoking status				< 0.001
Never (*n* = 2319; 49.7%)	2319 (76.4%)	0 (0.0%)	0 (0.0%)	
Former (*n* = 1256; 26.9%)	441 (14.5%)	454 (49.3%)	361 (50.8%)	
Current (*n* = 1093; 23.4%)	277 (9.1%)	467 (50.7%)	349 (49.2%)	
Pack-year^b^	0 [0, 0]	30 [30, 40]	50 [50, 65]	< 0.001
Lesion size (cm)^b^	2.7 [1.9, 4.0]	3.1 [2.1, 4.8]	3.2 [2.1, 5.2]	< 0.001
Lesion type				< 0.001
Part-solid (*n* = 224; 4.8%)	186 (6.1%)	30 (3.3%)	8 (1.1%)	
Solid (*n* = 4444; 95.2%)	2851 (93.9%)	891 (96.7%)	702 (98.9%)	
Location of lesion				0.002
Right upper lobe (*n* = 1260; 27.0%)	795 (26.2%)	244 (26.5%)	221 (31.1%)	
Right middle lobe (*n* = 314; 6.7%)	230 (7.6%)	51 (5.5%)	33 (4.6%)	
Right lower lobe (*n* = 1162; 24.9%)	761 (25.1%)	237 (25.7%)	164 (23.1%)	
Left upper lobe (*n* = 1100; 23.6%)	686 (22.6%)	229 (24.9%)	185 (26.1%)	
Left lower lobe (*n* = 832; 17.8%)	565 (18.6%)	160 (17.4%)	107 (15.1%)	
Lesion with spiculation				0.08
Absence (*n* = 3832; 82.1%)	2520 (83.0%)	746 (81.0%)	566 (79.7%)	
Presence (*n* = 836; 17.9%)	517 (17.0%)	175 (19.0%)	144 (20.3%)	
Lesion with open bronchus sign				< 0.001
Absence (*n* = 3701; 79.3%)	2344 (77.2%)	756 (82.1%)	601 (84.6%)	
Presence (*n* = 967; 20.7%)	693 (22.8%)	165 (17.9%)	109 (15.4%)	
Emphysema				< 0.001
Absence (*n* = 3948; 84.6%)	2886 (95.0%)	635 (68.9%)	427 (60.1%)	
Presence (*n* = 720; 15.4%)	151 (5.0%)	286 (31.1%)	283 (39.9%)	
Emphysema along needle tract				< 0.001
Absence (*n* = 4414; 94.6%)	2994 (98.6%)	816 (88.6%)	604 (85.1%)	
Presence (*n* = 254; 5.4%)	43 (1.4%)	105 (11.4%)	106 (14.9%)	
Operators’ experience				0.60
During less-experienced period (*n* = 2505; 53.7%)	1646 (54.2%)	486 (52.8%)	373 (52.5%)	
After sufficient experience (*n* = 2163; 46.3%)	1391 (45.8%)	435 (47.2%)	337 (47.5%)	
Procedure type				0.17
Fine needle aspiration only (*n* = 60; 1.3%)	44 (1.4%)	12 (1.3%)	4 (0.6%)	
Core needle biopsy (*n* = 4608; 98.7%)	2993 (98.6%)	909 (98.7%)	706 (99.4%)	
Needle indwelling time (min)^b^	7 [6, 8]	7 [6, 9]	7 [6, 8]	0.48

^a^ Smoking intensity was categorized as follows: (1) never-smokers or light smokers (≤ 20 pack-years), (2) moderate smokers (> 20 and ≤ 40 pack-years), and (3) heavy smokers (> 40 pack-years)

^b^ Median [interquartile range]

There were 1333 USPSTF-eligible (28.6%), 1694 NCCN-eligible (36.3%), and 819 NLST-eligible individuals (17.5%), respectively (Table S1).

### Smoking and diagnostic results

Malignancies were diagnosed in 3054 of 4668 PTNB procedures (65.4%), while a total of 1282 PTNB procedures yielded non-diagnostic pathologies (27.5%). The remaining 332 PTNBs provided specific benign pathologies (7.1%). Among non-diagnostic PTNBs, 10.1% (129/1282) were classified as undecidable, and false-negative results were found in 20.5% (236/1153) of the non-diagnostic PTNB procedures with decidable reference standards.

**Fig. 2:**
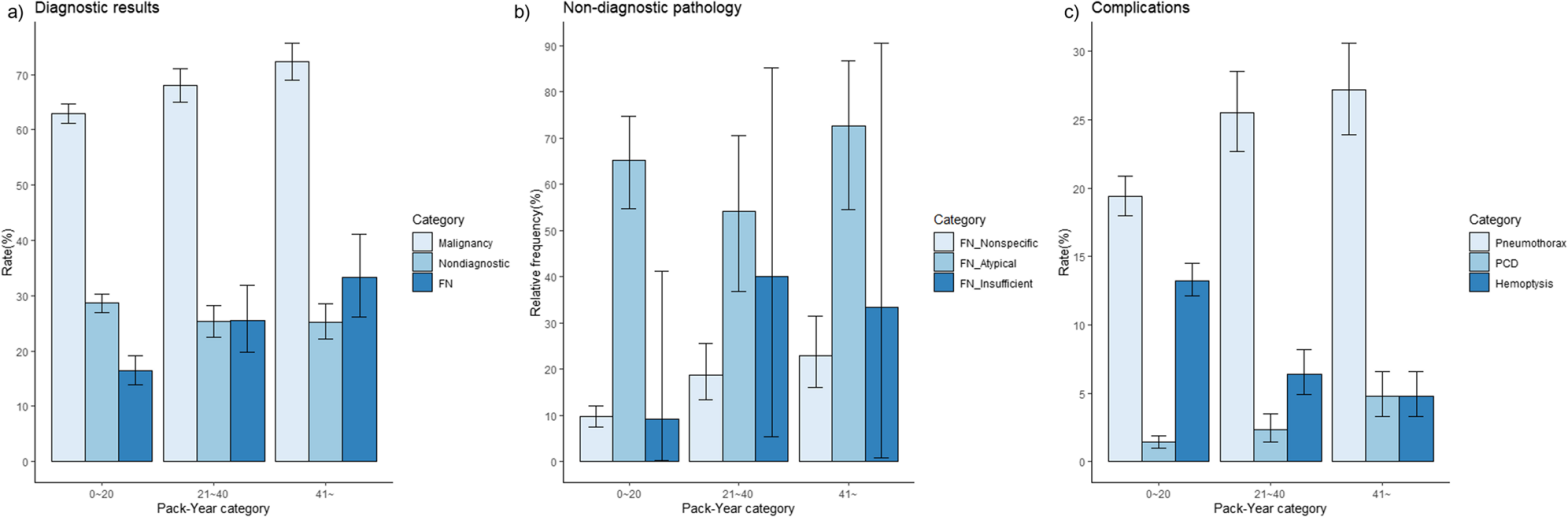
Rates or relative frequencies of events according to pack-year categories: (**a**) diagnostic results, (**b**) false-negative results in non-diagnostic pathologies, and (**c**) complications. **a** The Y-axis indicates diagnostic rates (malignancies, 63.0% [1913/3037] vs. 68.1% [627/921] vs. 72.4% [514/710]; non-diagnostic pathologies: 28.6% [870/3037] vs. 25.3% [233/921] vs. 25.2% [179/710]; false-negative results, 16.4% [128/779] vs. 25.5% [54/212] vs. 33.3% [54/162]). **b** The Y-axis indicates the relative frequency of false-negative results in three non-diagnostic pathology categories (non-specific benign pathologies, 9.7% [65/673] vs. 18.8% [32/170] vs. 23.0% [29/126]; atypical cells, 65.3% [62/95] vs. 54.1% [20/37] vs. 72.7% [24/33]; insufficient specimens, 9.1% [1/11] vs. 40.0% [2/5] vs. 33.3% [1/3]). **c** The Y-axis indicates complication rates (pneumothorax, 19.4% [590/3037] vs. 25.5% [235/921] vs. 27.2% [193/710]; pneumothorax requiring chest catheter drainage insertion, 1.4% [42/3037] vs. 2.3% [21/921] vs. 4.8% [34/710]; hemoptysis, 13.2% [402/3037] vs. 6.4% [59/921] vs. 4.8% [34/710])

The rates of malignancies and false-negative results in moderate and heavy smokers were higher than those in never or light smokers (malignancy, 63.0% vs. 68.1% vs. 72.4%, *p *= 0.005 and < 0.001, respectively; false-negative results, 16.4% vs. 25.5% vs. 33.3%, *p *= 0.003 and < 0.001, respectively) (Table 2, Fig. 2). The false-negative rates in non-diagnostic pathologies were lower in PTNBs performed by experienced operators (Table S2; 22.7% vs. 17.6%, *p *= 0.04). Detailed categories of non-diagnostic pathologies and final pathologies of false-negative PTNBs can be found in Table S3.

**Table 2:** Rates of events with confidence intervals according to smoking intensity

		Diagnostic results				Complications		
Pack-year		Diagnosis of malignancies	Non-diagnostic pathologies	False-negative results in non-diagnostic pathologies^a^	False-negative results in non-specific benign pathologies^a^	Pneumothorax	Pneumothorax requiring drainage catheter	Hemoptysis
Total	**Rates**	65.4%	27.5%	20.5%	13.0%	21.8%	2.1%	10.6%
	**95% CI**	64.0–66.8%	26.2–28.8%	18.2–22.9%	11.0–15.3%	20.6–23.0%	1.7–2.5%	9.7–11.5%
0–20	**Rates**	63.0%	28.6%	16.4%	9.7%	19.4%	1.4%	13.2%
	**95% CI**	61.2–64.7%	27.0–30.3%	13.9–19.2%	7.5–12.1%	18.0–20.9%	1.0–1.9%	12.1–14.5%
21–40	**Rates**	68.1%	25.3%	25.5%	18.8%	25.5%	2.3%	6.4%
	**95% CI**	65.0–71.1%	22.5–28.2%	19.8–31.9%	13.3–25.5%	22.7–28.5%	1.4–3.5%	4.9–8.2%
> 40	**Rates**	72.4%	25.2%	33.3%	23.0%	27.2%	4.8%	4.8%
	**95% CI**	69.0–75.7%	22.1–28.6%	26.1–41.2%	16.0–31.4%	23.9–30.6%	3.3–6.6%	3.3–6.6%
Pairwise comparison^b^	**0–20 vs. 21–40**	0.005	0.05	0.003	< 0.001	< 0.001	0.06	< 0.001
	**0–20 vs. > 40**	< 0.001	0.07	< 0.001	< 0.001	< 0.001	< 0.001	< 0.001
	**21–40 vs. > 40**	0.06	0.97	0.10	0.38	0.45	0.005	0.16

Abbreviations: *CI* confidence interval

^a^ From non-diagnostic pathologies or non-specific benign pathologies with decidable reference standards

^b^ If *p* value is less than 0.05/3 (= 0.0167), it was thought to be statistically significant

Current smoking status was an independent risk factor for a malignancy diagnosis (adjusted odds ratio [OR], 1.31; 95% CI: 1.02–1.69; *p *= 0.03) (Tables 3 and S4). Heavy smoking was associated with a higher rate of non-diagnostic pathologies (OR, 1.69; 95% CI: 1.19–2.40; *p *= 0.003). In addition, current smoking status and heavy smoking were independent risk factors for false-negative results in non-diagnostic pathologies, respectively (OR, 2.64 and 2.12, respectively; 95% CI: 1.32–5.28 and 1.17–3.92, respectively; *p = *0*.*006 and 0.02, respectively). Similarly, these factors contributed to false-negative results in non-specific benign pathologies (OR, 2.59 and 2.38, respectively; 95% CI: 1.16–5.72 and 1.19–4.90, respectively; *p = *0*.*02 and 0.02, respectively) (Tables 3 and S4). The effects of smoking on diagnostic outcomes were similar in PTNBs performed using the core needle biopsy technique and in sensitivity analyses (Tables S5 and S6).

**Table 3:** Effect of smoking on diagnostic results and complications of percutaneous transthoracic needle biopsy

	Adjusted OR	95% CI	*p* value
Diagnostic results			
Diagnosis of malignancies			
Former smoker	1.14	0.90–1.44	0.27
Current smoker	1.31	1.02–1.69	0.03
Moderate smoker: pack-year (> 20, ≤ 40)	1.17	0.94–1.46	0.17
Heavy smoker: pack-year (> 40)	1.22	0.96–1.56	0.11
Non-diagnostic pathologies^a^			
Former smoker	1.17	0.83–1.64	0.37
Current smoker	0.97	0.67–1.39	0.85
Moderate smoker: Pack-year (> 20, ≤ 40)	1.08	0.78–1.49	0.64
Heavy smoker: Pack-year (> 40)	1.69	1.19–2.40	0.003
False-negative results in non-diagnostic pathologies^b^			
Former smoker	1.72	0.89–3.30	0.10
Current smoker	2.64	1.32–5.28	0.006
Moderate smoker: pack-year (> 20, ≤ 40)	1.74	0.98–3.13	0.06
Heavy smoker: pack-year (> 40)	2.12	1.17–3.92	0.02
False-negative results in non-specific benign pathologies^c^			
Former smoker	1.65	0.76–3.54	0.20
Current smoker	2.59	1.16–5.72	0.02
Moderate smoker: pack-year (> 20, ≤ 40)	2.40	1.24–4.76	0.01
Heavy smoker: pack-year (> 40)	2.38	1.19–4.90	0.02
Complications			
Pneumothorax			
Former smoker	1.01	0.77–1.33	0.92
Current smoker	0.96	0.71–1.28	0.77
Moderate smoker: pack-year (> 20, ≤ 40)	1.24	0.96–1.59	0.10
Heavy smoker: pack-year (> 40)	1.33	1.01–1.74	0.04
Pneumothorax requiring catheter drainage			
Former smoker	1.49	0.72–2.98	0.27
Current smoker	1.66	0.77–3.43	0.18
Moderate smoker: pack-year (> 20, ≤ 40)	0.92	0.45–1.92	0.81
Heavy smoker: pack-year (> 40)	1.80	0.92–3.68	0.10
Hemoptysis			
Former smoker	0.88	0.61–1.26	0.48
Current smoker	0.64	0.42–0.96	0.04
Moderate smoker: pack-year (> 20, ≤ 40)	0.81	0.55–1.19	0.28
Heavy smoker: pack-year (> 40)	0.64	0.40–0.99	0.048

Abbreviations: *OR*, odds ratio; *CI*, confidence interval

Detailed results can be found in Table S4

^a^ From 4539 PTNB procedures with decidable reference standards

^b^ From 1153 non-diagnostic pathologies with decidable reference standards

^c^ From 969 non-specific benign pathologies with decidable reference standards

Lung cancer screening eligibility criteria were also associated with false-negative results in non-diagnostic pathologies (USPSTF eligibility, OR: 1.79, 95% CI: 1.16–2.76, *p *= 0.008; NCCN eligibility, OR: 2.17, 95% CI: 1.38–3.45, *p* < 0.001) and non-specific benign pathologies (USPSTF eligibility, OR: 2.17, 95% CI: 1.31–3.63, *p *= 0.003; NCCN eligibility, OR: 2.50, 95% CI: 1.48–4.35, *p* < 0.001; NLST eligibility, OR: 1.79, 95% CI: 1.09–2.89, *p *= 0.02) (Tables 4 and S7).

**Table 4:** Abbreviated results of multivariable logistic regression analysis with backward elimination for the associations between lung cancer screening eligibility and the results of percutaneous transthoracic needle biopsy

	Adjusted OR	95% CI	*p* value
Diagnostic results			
Diagnosis of malignancies			
USPSTF eligibility	1.39	1.18–1.64	< 0.001
NCCN eligibility	1.56	1.32–1.85	< 0.001
NLST eligibility	1.31	1.09–1.58	0.004
Non-diagnostic pathologies^a^			
USPSTF eligibility	N/I		
NCCN eligibility	1.32	1.07–1.65	0.01
NLST eligibility	N/I		
False-negative results in non-diagnostic pathologies^b^			
USPSTF eligibility	1.79	1.16–2.76	0.008
NCCN eligibility	2.17	1.38–3.45	< 0.001
NLST eligibility	1.53	0.96–2.41	0.07
False-negative results in non-specific benign pathologies^c^			
USPSTF eligibility	2.17	1.31–3.63	0.003
NCCN eligibility	2.50	1.48–4.35	< 0.001
NLST eligibility	1.79	1.09–2.89	0.02
Complications			
Pneumothorax			
USPSTF eligibility	1.23	1.05–1.45	0.01
NCCN eligibility	1.30	1.11–1.51	0.001
NLST eligibility	N/I		
Pneumothorax requiring catheter insertion			
USPSTF eligibility	N/I		
NCCN eligibility	N/I		
NLST eligibility	N/I		
Hemoptysis			
USPSTF eligibility	0.78	0.58–1.05	0.10
NCCN eligibility	0.72	0.54–0.97	0.03
NLST eligibility	0.68	0.47–0.96	0.03

Abbreviations: *OR*, odds ratio; *CI*, confidence interval; *USPSTF*, US Preventive Service Task Force; *NCCN*, National Comprehensive Cancer Network; *NLST*, National Lung Screening Trial; *N/I,* not included in final logistic regression model

Detailed results can be found in Table S7

^a^ From 4539 PTNB procedures with decidable reference standards

^b^ From 1153 non-diagnostic pathologies with decidable reference standards

^c^ From 969 non-specific benign pathologies with decidable reference standards

**Fig. 3:**
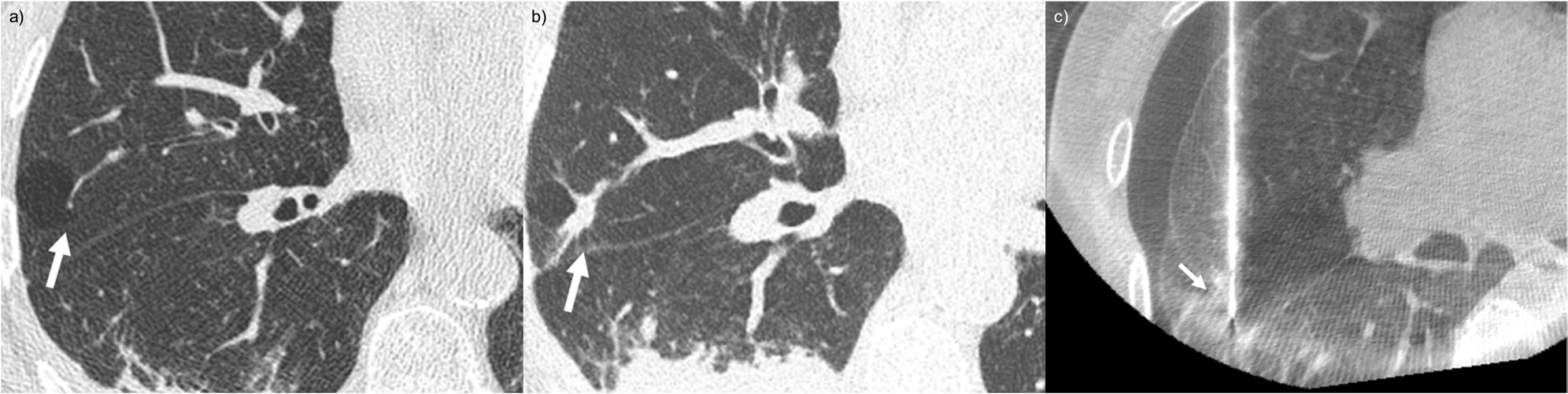
False-negative percutaneous transthoracic needle biopsy (PTNB) in a heavy smoker: **a**, **b**, **c** A 71-year-old man (current smoker; 50 pack-years) with a 1.3-cm growing lung nodule in the right middle lobe for 4 years underwent PTNB for the diagnosis. The pathologic specimen was non-neoplastic lung parenchyma. Pneumothorax occurred during the procedure (**c**), and a drainage catheter was inserted after PTNB because of the substantial amount of pneumothorax (not presented). Adenocarcinoma was diagnosed on repeated PTNB

**Fig. 4:**
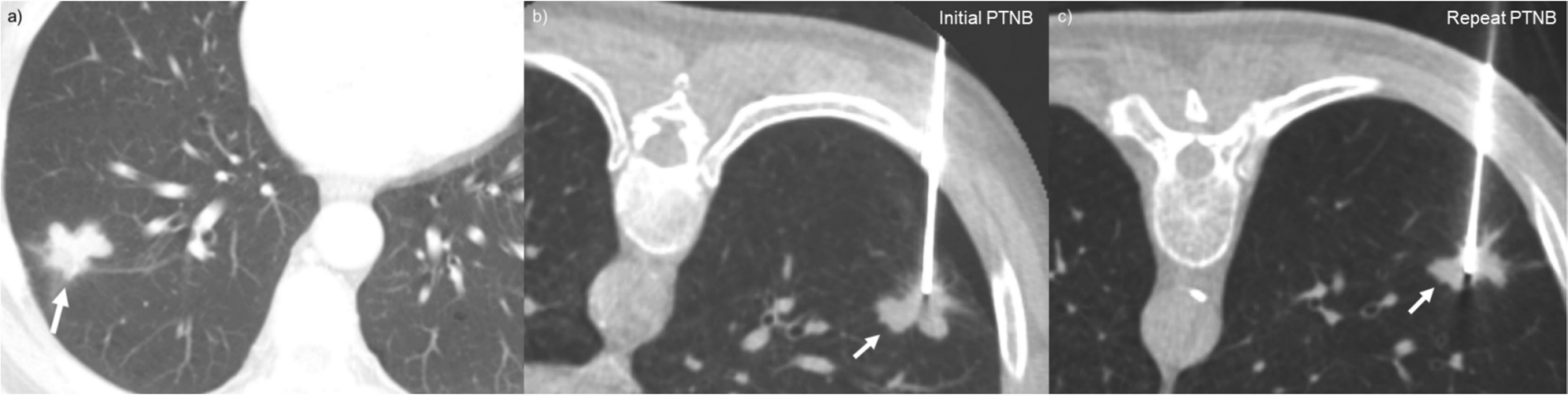
False-negative percutaneous transthoracic needle biopsy (PTNB) in a heavy smoker: **a** A 66-year-old man (current smoker; 45 pack-years) with a 2.7-cm incidental lung nodule in the right lower lobe underwent PTNB for the diagnosis. **b** The positioning of the tip of the core needle was thought to be appropriate based on the procedural images, and the operators obtained tissue samples. However, the pathologic result was non-diagnostic (necrosis with foamy histiocyte collection and multinucleated giant cells), and pneumothorax developed during the procedure. The amount of pneumothorax increased during follow-up, and a drainage catheter was placed to alleviate the symptom (not presented). **c** As the pre-procedural CT images were highly suggestive of lung cancer, the patient underwent repeated PTNB. On the procedural images, the position of the core needle tip was not substantially different from the previous one, but squamous cell carcinoma was confirmed on repeated PTNB

**Fig. 5:**
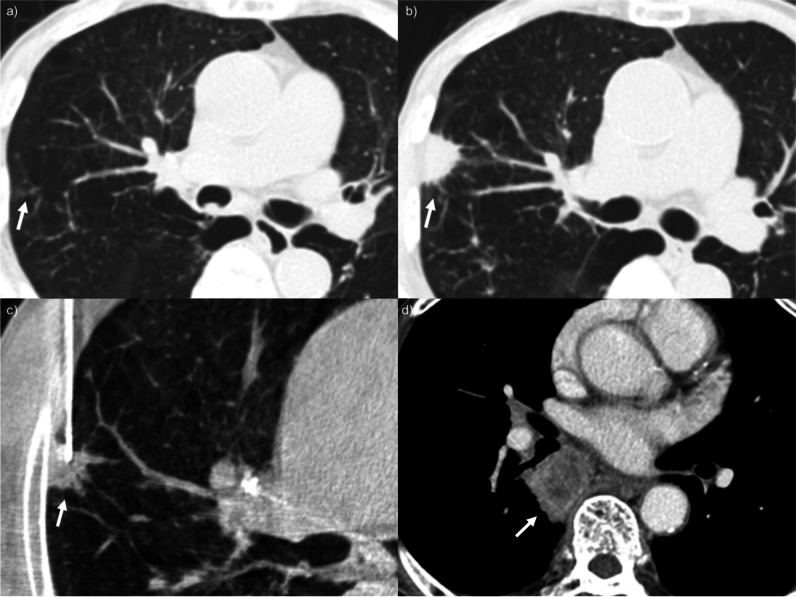
False-negative percutaneous transthoracic needle biopsy (PTNB) in a heavy smoker: **a**, **b** A 76-year-old man (current smoker; 81 pack-years) with a growing 2.7-cm lung nodule in the right upper lobe for 8 months underwent PTNB for the diagnosis. **c** The pathologic result was non-diagnostic (fibrosis and inflammation), and pneumothorax was noted on follow-up chest radiographs (not presented). **d** Endobronchial ultrasound-guided transbronchial needle biopsy was performed for the left upper paratracheal and both lower paratracheal and subcarinal lymph nodes. Metastatic adenocarcinoma was confirmed at the subcarinal lymph node

Representative cases of false-negative PTNBs are presented in Figs. 3, 4, and 5.

### Smoking and complications

The rates of pneumothorax, pneumothorax requiring chest drainage catheter insertion, and hemoptysis were 21.8% (1018/4668), 2.1% (97/4668), and 10.6% (495/4668), respectively. The rates of pneumothorax were higher in moderate and heavy smokers than in the reference group (19.4% vs. 25.5% vs. 27.2%, *p* < 0.001 and < 0.001, respectively), whereas the rate of hemoptysis was lower in moderate and heavy smokers (13.2% vs. 6.4% vs. 4.8%, *p *< 0.001 and < 0.001, respectively) (Table 2, Fig. 2). The complication rates were not different according to the operators’ experience (*p* > 0.05) (Table S2).

Pneumothorax during or after PTNB procedures occurred more frequently in heavy smokers (OR, 1.33; 95% CI: 1.01–1.74, *p = *0*.*04), but the requirement for chest drainage catheter insertion was not associated with heavy smoking (*p = *0*.*10). The development of hemoptysis was significantly less common in current smokers (OR, 0.64; 95% CI: 0.42–0.96, *p = *0*.*04) and heavy smokers (OR, 0.64; 95% CI: 0.40–0.99, *p = *0*.*048) (Tables 3 and S4). The effects of smoking on complications were similar in PTNBs performed using the core needle biopsy technique, but the association between smoking history and hemoptysis was borderline significant (current smoking, *p *= 0.05; heavy smoking, *p *= 0.06; Table S5). The results of the sensitivity analyses can be found in Table S6.

Eligibility, according to the USPSTF and NCCN screening criteria, was a risk factor for pneumothorax (OR, 1.23 and 1.30; 95% CI: 1.05–1.45 and 1.11–1.51; *p = *0*.*01 and 0.001, respectively). Conversely, NCCN and NLST screening eligibility showed a protective association against PTNB-associated hemoptysis (OR, 0.72 and 0.68; 95% CI: 0.54–0.97 and 0.47–0.96; *p = *0*.*03 and 0.03, respectively) (Tables 4 and S7).

## Discussion

**Fig. 6:**
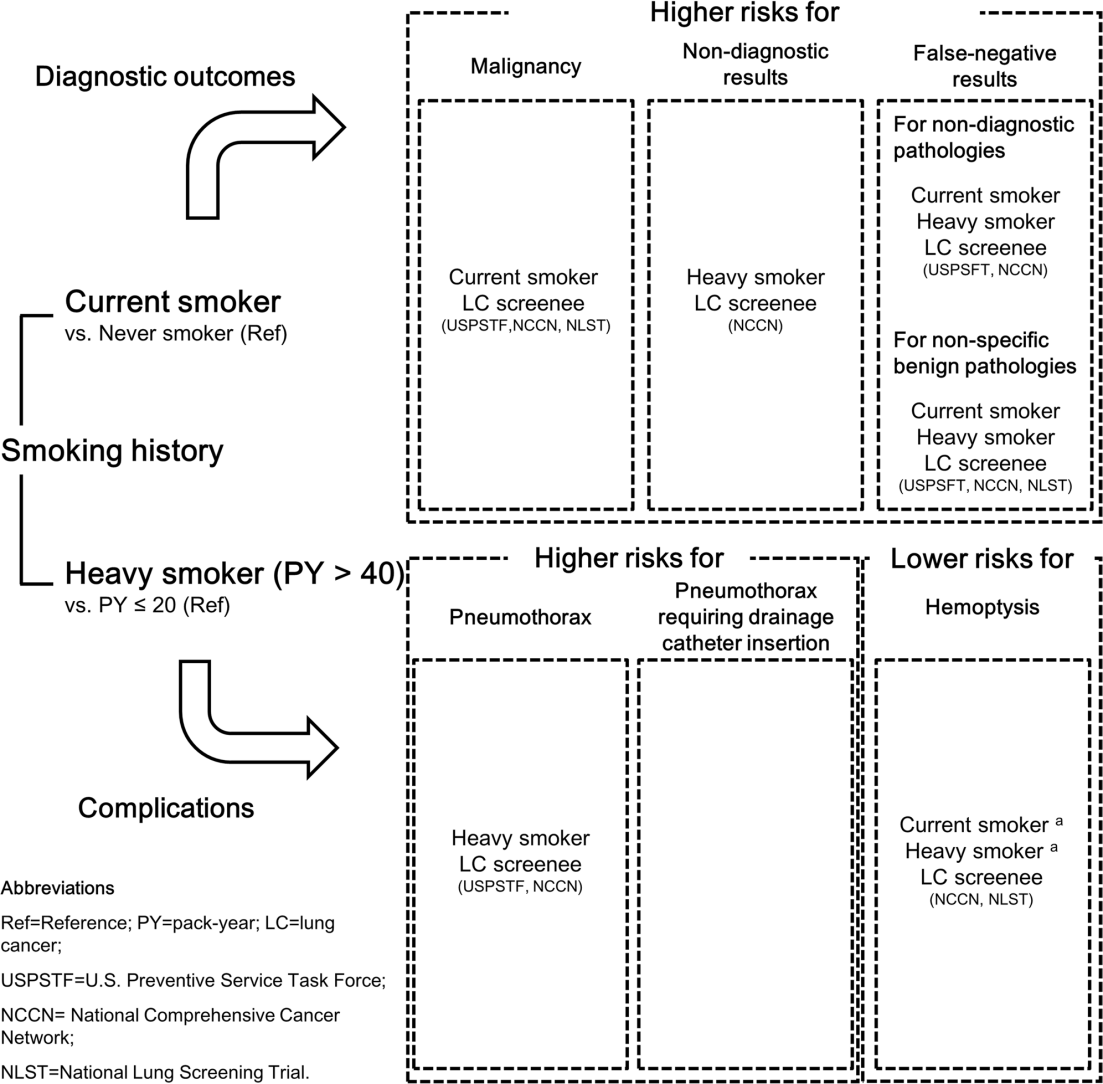
Associations between current or heavy smoking history and the outcomes of percutaneous transthoracic needle biopsy. ^a^Variables with borderline statistical significance in the subgroup analysis for percutaneous transthoracic needle biopsy using the core needle biopsy technique

This retrospective single-institutional study demonstrated that smoking had a significant impact on the outcomes of PTNB, and a summary of the results of this study can be found in Fig. 6. Current smoking was associated with higher likelihoods of a malignancy diagnosis (adjusted OR, 1.31) and false-negative results in non-diagnostic PTNBs (OR, 2.64). Heavy smoking was an independent risk factor for false-negative results (OR, 2.12). Regarding complications, heavy smoking was a significant factor associated with pneumothorax (OR, 1.33) and hemoptysis (OR, 0.64), while current smoking was another protective factor against hemoptysis (OR, 0.64). Furthermore, lung cancer screening-eligible individuals showed similar patterns.

The most important finding of this study is that false-negative rates were higher in heavy smokers or lung cancer screening-eligible patients with non-diagnostic PTNBs. Specifically, both current smoking status and heavy smoking history were independently associated with higher rates of false-negative results in biopsies with non-specific benign pathologies. These results indicate that caution is warranted due to the higher rates of false-negative results when PTNB procedures yield non-diagnostic results in current or heavy smokers. In such cases, close monitoring of pulmonary lesions with non-specific benign results is advised, and more rigorous diagnostic procedures (e.g., repeated biopsy) may be necessary if clinical or imaging findings are inconsistent with the biopsy results.

The adequacy of PTNB specimens was routinely assessed by experienced thoracic radiologists during the study period, but the presence of on-site pathologists during PTNBs could potentially reduce the incidence of false-negative results. However, it is also expected that the rates of pneumothorax and the requirement for chest catheter drainage would also increase because of the prolonged needle indwelling time. Thus, even when PTNBs are performed in heavy smokers, the availability of rapid on-site evaluation of PTNB specimens needs to be tailored based on the operators’ experience and institutional resources. The characteristics of PTNB specimens (length or thickness) might be associated with the development of complications, but it was not possible to quantify these factors during PTNB procedures, and the association between them was beyond the scope of this study.

The results of higher non-diagnostic and false-negative rates in heavy smokers may partly originate from targeting error due to limited respiratory cooperation in heavy smokers. However, it could be speculated that the contribution of technical failure to the results of this study might be insignificant, as the operators determined the appropriateness of tip location and gross morphology of specimens during the PTNB procedures.

Alternatively, these results might be attributed to the tumor microenvironment [31, 32]. Previous studies on the role of smoking in the tumor microenvironment have shown conflicting results. For example, one study reported that the type of infiltrating inflammatory cells around adenocarcinomas could vary according to smoking habits [33], while another study found no correlation between the level of tumor-infiltrating lymphocytes and smoking status [34]. Thus, the tumor microenvironment needs to be investigated further to identify whether smoking truly influences the tumor microenvironment and subsequently, false-negative results of PTNBs.

In terms of PTNB-associated complications, lower hemoptysis rates were consistently found as pack-years increased. In a previous study [11], a history of smoking (vs. never-smoking) showed borderline statistical significance with the occurrence of hemoptysis (*p = *0*.*06). In this study, ever-smokers were stratified into former and current smokers, and the intensity of smoking was additionally investigated. Both current smoking (OR, 0.64) and heavy smoking (OR, 0.64) were independently associated with hemoptysis; these associations could be explained by pulmonary vascular remodeling and hypoxic pulmonary vasoconstriction [35]. In contrast, pneumothorax was associated with heavy smokers (OR, 1.33), even after adjusting for the presence of emphysema, which is one of the most important risk factors for PTNB-associated pneumothorax. These results suggest that the occurrence of pneumothorax should be kept in mind before performing PTNB in heavy smokers, regardless of the presence of emphysema. Pulmonary function might mediate the relationship between smoking intensity and complications, but the results of pulmonary function tests were not available for all patients. Nevertheless, we believe that patients’ ability to hold their breath during PTNBs could serve as a direct proxy for PTNB-associated complications, particularly in the absence of pulmonary function tests.

This single-institutional retrospective study had some limitations. First, unidentified bias might exist because of the retrospective study design. In addition, the results of this study were not validated in an external dataset, which could contain data from settings where PTNB procedures were performed in different manners, especially in terms of the biopsy-guiding modality. Nonetheless, previous studies have suggested that the diagnostic results and complication risks under CT guidance did not differ from those under cone-beam CT guidance [10, 11]. It is also expected that patients’ comorbidities could affect their respiratory cooperation during PTNBs, which might also be a confounder in this study. However, PTNBs were usually halted in patients with poor respiratory cooperation. Second, pack-years were collected by self-reporting, which can result in variability and be affected by under-reporting issues [36]. Third, PTNB was performed on the basis of shared decision-making between clinicians and radiologists, and the indications for PTNB may vary at other institutions. The results of this study may not be generalizable to all settings. A prospective multicenter study would be needed to provide more robust evidence regarding the relationship between smoking and biopsy outcomes.

In conclusion, smoking history was associated with the diagnostic results and complications of PTNB procedures. Non-diagnostic pathologies should be interpreted cautiously in current smokers, heavy smokers, or lung cancer screening-eligible individuals. It is important to consider smoking history before PTNB procedures to predict and manage procedure-related complications.

## Supplementary Information

Below is the link to the electronic supplementary material.

Supplementary file1 (PDF 527 KB)

## Abbreviations

CI: Confidence interval
NCCN: National Comprehensive Cancer Network
NLST: National Lung Screening Trial
OR: Odds ratio
PTNB: Percutaneous transthoracic needle biopsy
USPSTF: US Preventive Services Task Force

## Declarations

### Guarantor

The scientific guarantor of this publication is Pf. Soon Ho Yoon.

### Statistics and biometry

The Medical Research Collaborating Center of Seoul National University Hospital (MRCC, https://mrcc.snuh.org) reviewed statistical analyses of this study.

### Study subjects or cohorts overlap

Data from some patients were previously reported, but none of those studies had an exact overlap with the study patients or focused on the association between smoking and PTNB.

### Methodology

- retrospective
- case-control study
- performed at one institution
