# Supplementary material for: Effect of smoking on the diagnostic results and complication rates of percutaneous transthoracic needle biopsy
Source: Eur Radiol. 2024 Mar 25;34(10):6514–26. doi: 10.1007/s00330-024-10705-8 (PMC11399209; doi:10.1007/s00330-024-10705-8)
Supplement: Supplementary file 1 — Supplementary file1 (PDF 527 KB) [file 330_2024_10705_MOESM1_ESM.pdf]

Effect of smoking on the diagnostic results and complication rates of percutaneous transthoracic needle biopsy

ELECTRONIC SUPPLEMENTARY MATERIAL

Contents

Supplementary Texts

Method S1. Details of Percutaneous Transthoracic Needle Biopsy Procedures

Method S2. Details of Collected Data

Method S3. Details of the Logistic Regression Analysis

Supplementary Tables

Table S1. Characteristics of Lung Cancer Screening-Eligible Individuals

Table S2. Outcomes of Percutaneous Transthoracic Needle Biopsy Procedures According to the Operators' Experience

Table S3. Detailed Results of Percutaneous Transthoracic Needle Biopsy with False-Negative Results

Table S4. Detailed Results of Logistic Regression Analysis After Adjusting for Covariates

Table S5. Detailed Results of Logistic Regression Analysis After Adjusting for Covariates: Subgroup Analysis for Percutaneous Transthoracic Needle Biopsy with the Core Needle Biopsy Technique

Table S6. Results of Multivariable Logistic Regression with Backward Elimination in All Patients

Table S7. Results of Multivariable Logistic Regression with Backward Elimination Regarding the Effect of Lung Cancer Screening Eligibility on Percutaneous Transthoracic Needle Biopsy

Supplementary Text

Method S1. Details of Percutaneous Transthoracic Needle Biopsy Procedures

All the percutaneous transthoracic needle biopsy (PTNB) procedures were performed either by board-certified thoracic radiologists under the supervision of attending thoracic radiologists or attending thoracic radiologists alone. The majority of the PTNB procedures were performed by thoracic radiologists taking part in fellowship training, who started their training in March or May of each year. There were usually 2 or 3 thoracic radiologists in fellowship training each year, and they usually rotated thoracic intervention schedules (including PTNB procedures) for 2 to 3 months. Attending thoracic radiologists performed the minority of PTNBs. Two cone-beam CT machines were used during the study period (Axiom Artis dTA/VB30, Siemens; Allura Xper FD20, Philips Healthcare).

If there were multiple candidates for PTNBs, the target lesion was selected by the operators with consideration of the yield and potential complication risks; therefore, peripherally located lesions, larger lesions, or lesions not abutting vessels or emphysematous cysts were usually selected. The PTNB technique (coaxial technique vs. fine-needle aspiration only) and needle size were chosen by the operators [1-4]. After introducing the needle, cone-beam CT scanning was performed to identify whether the tip of the needle was located properly, and if needed, the needle tip was adjusted under cone-beam fluoroscopic guidance to obtain optimal pathologic specimens. On-site pathologists did not attend the PTNB procedures, and the operators chose the number of tissue samples based on the adequacy of the PTNB specimens according to factors such as the specimens' length, thickness, and color. The needle indwelling time (fine-needle aspiration: time interval between initial needle insertion and final needle removal; core needle biopsy: time interval between the insertion and removal of core needle)

was routinely recorded by the radiology technicians.

Method S2. Details of Collected Data

Information on target lesions, such as the lesion location, lesion size, and lesion type (solid vs. part-solid), was collected from standardized PTNB reports. Operators assessed the presence of emphysema based on preprocedural CT findings. Information on the PTNB procedures, including the pleura-to-target distance, the position during the PTNB procedure, biopsy needle size, the number of pleural passages, the number of tissue samples, and needle indwelling time, was also collected in the same manner. In addition, the presence of emphysema along the needle tract, the presence of the open bronchus sign in the target lesion, and the presence of spiculation at the target lesion were also identified based on preprocedural CT findings.

The operators recorded complications (pneumothorax, pneumothorax requiring chest drainage catheter insertion, or hemoptysis) in PTNB reports by reviewing post-PTNB follow-up images and electronic medical records. The development of immediate pneumothorax or non-immediate pneumothorax during the admission period was defined as the presence of pneumothorax. The first author (W.H.L) reviewed procedural and post-procedural images to identify pneumothorax. A study coordinator curated the pathologic results of PTNB specimens from patients' electronic medical records.

Method S3. Details of the Logistic Regression Analysis

This study considered the operators' proficiency in PTNB procedures and the difficulty of PTNB procedures because these factors might be associated with the outcomes of the PTNBs. In detail, reflecting the annual schedule of fellowship training, the operators were thought to have less experience in PTNBs if the procedures were performed between March and August of each year, whereas the PTNBs being performed between September and February of each year were considered to be performed by thoracic radiologists with sufficient experience in PTNBs [5]. The analyses adjusted for the proficiency of the operators, the presence of emphysema along the needle tract (one of the major risk factors for pneumothorax requiring catheter drainage), the presence of the open bronchus sign in the target lesion (one of the major risk factors for hemoptysis), and needle indwelling time (a surrogate for the difficulty of the PTNB procedure and the operators' experience) [1, 5, 6].

To focus on the effect of smoking on the diagnostic results and complications of PTNB procedures, logistic regression was performed with adjustment for the following covariates based on previous studies: 1) diagnosis of malignancies: age, sex, location of the lesion, size, lesion type (solid vs. part-solid), the presence of spiculation, the presence of emphysema, the presence of emphysema along the needle tract, the presence of the open bronchus sign, needle indwelling time, and the operators' experience (during the less-experienced period vs. after sufficient experience) [1, 5-7]; 2) non-diagnostic pathologies: age (> 65 years vs. ≤ 65 years), sex, size (≤ 1 cm, > 1 cm and ≤ 2 cm, > 2 cm and ≤ 3 cm, or > 3 cm), lesion type (solid vs. part-solid), procedure with fine-needle aspiration only, the number of tissue samples with a cutting needle (≥ 3 vs. < 3), position during the PTNB procedure (supine, prone, or lateral decubitus), the presence of hemoptysis, the final diagnosis (malignancy vs. benign), the

presence of emphysema along the needle tract, the presence of the open bronchus sign, needle indwelling time, and the operators' experience (during the less-experienced period vs. after sufficient experience) [1, 5, 6, 8, 9]; 3) false-negative results in non-diagnostic pathologies: age (> 65 years vs. ≤ 65 years), sex, the presence of emphysema, location of the lesion, lesion type (solid vs. part-solid), the category of non-diagnostic pathologies (non-specific benign, atypical cells, or insufficient for diagnosis), the presence of emphysema along the needle tract, the presence of the open bronchus sign, needle indwelling time, and the operators' experience (during the less-experienced period vs. after sufficient experience) [1, 5, 6, 9]; 4) false-negative results in non-specific benign pathologies: age (> 65 years vs. ≤ 65 years), sex, the presence of emphysema, lesion type (solid vs. part-solid), the presence of emphysema along the needle tract, the presence of the open bronchus sign, needle indwelling time, and the operators' experience (during the less-experienced period vs. after sufficient experience) [1, 5, 6, 9]; 5) pneumothorax: age (≥ 60 years vs. < 60 years), sex, location of the lesion, size (> 2 cm vs. ≤ 2 cm), lesion type (solid vs. part-solid), the presence of emphysema, pleura-to-target distance (≤ 2 cm, > 2 cm and ≤ 4 cm, or > 4 cm), the number of pleural passages (1, 2, or > 2), the presence of emphysema along the needle tract, the presence of the open bronchus sign, needle indwelling time, and the operators' experience (during the less-experienced period vs. after sufficient experience) [1, 5, 6, 10], 6) pneumothorax requiring chest drainage catheter insertion: age (≥ 60 years vs. < 60 years), size (> 2 cm vs. ≤ 2 cm), lesion type (solid vs. part-solid), the presence of emphysema, pleura-to-target distance (≤ 2 cm, > 2 cm and ≤ 4 cm, or > 4 cm), the number of pleural passages (1, 2, or > 2), the presence of emphysema along the needle tract, the presence of the open bronchus sign, needle indwelling time, and the operators' experience (during the less-experienced period vs. after sufficient experience) [1, 5, 6, 10]; and 7) hemoptysis: sex, size (> 2 vs. ≤ 2 cm), lesion type (solid vs. part-solid), the presence of

emphysema, pleura-to-target distance (≤ 2 cm, > 2 cm and ≤ 4 cm, or > 4 cm), the presence of emphysema along the needle tract, the presence of the open bronchus sign, needle indwelling time, and the operators' experience (during the less-experienced period vs. after sufficient experience) [1, 5, 6, 10].

In multivariable logistic regression analysis with backward elimination (as sensitivity analyses), the full models included the following variables: 1) diagnosis of malignancies: smoking status (never, former, or current smokers), pack-year category (≤ 20, > 20 and ≤ 40, or > 40), age (> 65 years vs. ≤ 65 years), sex, location of the lesion (upper or middle lobe vs. lower lobe), size (> 2 cm vs. ≤ 2 cm), lesion type (solid vs. part-solid), the presence of emphysema, the presence of spiculation, the presence of emphysema along the needle tract, the presence of the open bronchus sign, needle indwelling time, and the operators' experience (during the less-experienced period vs. after sufficient experience); 2) non-diagnostic pathologies: smoking status (never, former, or current smokers), pack-year category (≤ 20, > 20 and ≤ 40, or > 40), age (> 65 years vs. ≤ 65 years), sex, location of the lesion (upper or middle lobe vs. lower lobe), size (> 2 cm vs. ≤ 2 cm), lesion type (solid vs. part-solid), the presence of emphysema, pleura-to-target distance (≤ 2 cm, > 2cm and ≤ 4 cm, > 4 cm), position during the PTNB procedure (supine, prone, or lateral decubitus), needle size (18G vs. smaller than 18G), procedure with fine-needle aspiration only, the number of pleural passages (1 vs. multiple), the number of tissue samples with a cutting needle (< 3 vs. ≥ 3 times), the presence of pneumothorax, the presence of hemoptysis, the final diagnosis (malignancy vs. benign), the presence of emphysema along the needle tract, the presence of the open bronchus sign, needle indwelling time, and the operators' experience (during the less-experienced period vs. after sufficient experience); 3) false-negative results in non-diagnostic pathologies: smoking status

(never, former, or current smokers), pack-year category (≤ 20, > 20 and ≤ 40, or > 40), age (> 65 years vs. ≤ 65 years), sex, location of the lesion (upper or middle lobe vs. lower lobe), size (> 2 cm vs. ≤ 2 cm), lesion type (solid vs. part-solid), the presence of emphysema, pleura-to-target distance (≤ 2 cm, > 2 cm and ≤ 4 cm, or > 4 cm), position during the PTNB procedure (supine, prone, lateral decubitus), needle size (18G vs. smaller than 18G), procedure with fine-needle aspiration only, the number of pleural passages (1 vs. multiple), the number of tissue samples with a cutting needle (< 3 vs. ≥ 3), the presence of pneumothorax, the presence of hemoptysis, the category of non-diagnostic pathologies (non-specific benign pathologies, atypical cells, or insufficient for diagnosis) the presence of emphysema along the needle tract, the presence of the open bronchus sign, needle indwelling time, and the operators' experience (during the less-experienced period vs. after sufficient experience); 4) pneumothorax and pneumothorax requiring chest drainage catheter insertion: smoking status (never, former, or current smokers), pack-year category (≤ 20, > 20 and ≤ 40, or > 40), age (> 65 years vs. ≤ 65 years), sex, location of the lesion (upper or middle lobe vs. lower lobe), size (> 2 cm vs. ≤ 2 cm), lesion type (solid vs. part-solid), the presence of emphysema, pleura-to-target distance (≤ 2 cm, > 2cm and ≤ 4 cm, or > 4 cm), position during the PTNB procedure (supine, prone, or lateral decubitus), needle size (18G vs. smaller than 18G), procedure with fine-needle aspiration only, the number of pleural passages (1 vs. multiple), the number of tissue samples with a cutting needle (< 3 vs. ≥ 3), the presence of hemoptysis, the presence of emphysema along the needle tract, the presence of the open bronchus sign, needle indwelling time, and the operators' experience (during the less-experienced period vs. after sufficient experience); 5) hemoptysis: smoking status (never, former, current smokers), pack-year category (≤ 20, > 20 and ≤ 40, or > 40), age (> 65 years vs. ≤ 65 years), sex, location of the lesion (upper or middle lobe vs. lower lobe), size (> 2 cm vs. ≤ 2 cm), lesion type (solid vs. part-solid), the presence of

emphysema, pleura-to-target distance (≤ 2 cm, > 2cm and ≤ 4 cm, or > 4 cm), position during the PTNB procedure (supine, prone, or lateral decubitus), needle size (18G vs. smaller than 18G), procedure with fine-needle aspiration only, the number of pleural passages (1 vs. multiple), the number of tissue samples with a cutting needle (< 3 vs. ≥ 3), the presence of pneumothorax, the presence of emphysema along the needle tract, the presence of the open bronchus sign, needle indwelling time, and the operators' experience (during the less-experienced period vs. after sufficient experience).

The associations of lung cancer screening eligibility with the diagnostic results and complication rates of PTNB procedures were also explored: 1) U.S. Preventive Services Task Force (USPSTF) lung cancer screening eligibility (ever-smoker, age of 50 to 80 years with at least a 20 pack-year smoking history, current smoker or former smoker having quit smoking within the past 15 years) [11]; National Comprehensive Cancer Network (NCCN) lung cancer screening eligibility (ever-smoker, age equal to or more than 50 years with at least a 20 pack-year smoking history) [12]; and National Lung Screening Trial (NLST) eligibility (ever-smoker, age of 55 to 74 years with at least a 30 pack-year smoking history, current smoker or former smoker having quit smoking within the past 15 years) [13]. Patients whose cessation duration could not be documented through their electronic medical records were not considered eligible for screening according to the USPSTF and NLST criteria. Since eligibility for lung cancer screening was determined in terms of age and pack-years, logistic regression was performed without adjusting for those covariates to explore the effect of lung cancer screening eligibility on PTNB procedures.

References

1. Kim H, Park CM, Yoon SH et al (2018) Open Bronchus Sign on CT: A Risk Factor for Hemoptysis after Percutaneous Transthoracic Biopsy. Korean J Radiol 19:880-887

2. Hwang EJ, Kim H, Park CM, Yoon SH, Lim HJ, Goo JM (2018) Cone beam computed tomography virtual navigation-guided transthoracic biopsy of small (≤ 1 cm) pulmonary nodules: impact of nodule visibility during real-time fluoroscopy. Br J Radiol 91:20170805

3. Hwang EJ, Park CM, Yoon SH, Lim HJ, Goo JM (2018) Risk factors for haemoptysis after percutaneous transthoracic needle biopsies in 4,172 cases: Focusing on the effects of enlarged main pulmonary artery diameter. Eur Radiol 28:1410-1419

4. Kim MG, Yang BR, Park CM, Yoon SH (2022) Preoperative percutaneous needle lung biopsy techniques and ipsilateral pleural recurrence in stage I lung cancer. Eur Radiol 32:2683-2692

5. Ahn SY, Park CM, Yoon SH, Kim H, Goo JM (2019) Learning Curve of C-Arm Cone-beam Computed Tomography Virtual Navigation-Guided Percutaneous Transthoracic Needle Biopsy. Korean J Radiol 20:844-853

6. Kim JI, Park CM, Lee SM, Goo JM (2015) Rapid needle-out patient-rollover approach after cone beam CT-guided lung biopsy: effect on pneumothorax rate in 1,191 consecutive patients. Eur Radiol 25:1845-1853

7. McWilliams A, Tammemagi MC, Mayo JR et al (2013) Probability of cancer in pulmonary nodules detected on first screening CT. N Engl J Med 369:910-919

8. Lee KH, Lim KY, Suh YJ et al (2019) Diagnostic Accuracy of Percutaneous Transthoracic Needle Lung Biopsies: A Multicenter Study. Korean J Radiol 20:1300-1310

9. Lee KH, Lim KY, Suh YJ et al (2019) Nondiagnostic Percutaneous Transthoracic Needle Biopsy of Lung Lesions: A Multicenter Study of Malignancy Risk. Radiology 290:814-823

10. Yoon SH, Park CM, Lee KH et al (2019) Analysis of Complications of Percutaneous Transthoracic Needle Biopsy Using CT-Guidance Modalities In a Multicenter Cohort of 10568 Biopsies. Korean J Radiol 20:323-331

11. US Preventive Services Task Force; Krist AH, Davidson KW, Mangione CM et al (2021) Screening for Lung Cancer: US Preventive Services Task Force Recommendation Statement. JAMA 325:962-970

12. Wood DE, Kazerooni EA, Aberle D et al (2022) NCCN Guidelines® Insights: Lung Cancer Screening, Version 1.2022. J Natl Compr Canc Netw 20:754-764

13. National Lung Screening Trial Research Team; Aberle DR, Adams AM, Berg CD et al (2011) Reduced lung-cancer mortality with low-dose computed tomographic screening. N Engl J Med 365:395-409

Supplementary Tables

Table S1. Characteristics of Lung Cancer Screening-Eligible Individuals

	USPSTF eligible	NCCN eligible	NLST eligible
**Percentage of eligible patients**	28.6% [1333/4668]	36.3% [1694/4668]	17.5% [819/4668]
**Percentage of men**	97.6% [1301/1333]	97.5% [1651/1694]	98.9% [810/819]
**Age (years)**[a]	67 [61, 73]	69 [62, 75]	66 [61, 70]
**Pack-year**[a]	40 [30, 50]	40 [30, 50]	40 [40, 50]
**Smoking cessation duration (years)**[a]	1 [1, 3]	0 [0, 10][b]	0 [0, 2]

Abbreviations: USPSTF=U.S. Preventive Service Task Force, NCCN=National Comprehensive Cancer Network, NLST=National Lung Screening Trial.

[a] Median [interquartile range].

[b] From 1651 patients with documented cessation duration.

Table S2. Outcomes of Percutaneous Transthoracic Needle Biopsy Procedures According to the Operators' Experience

	During less-experienced period	After sufficient experience	*p*-value
**Diagnosis of malignancies**	65.1% (1632/2505)	65.7% (1422/2163)	0.69
**Non-diagnostic pathologies**	28.6% (717/2505)	26.1% (565/2163)	0.06
**False-negative results in non-diagnostic pathologies**[a]	22.7% (146/642)	17.6% (90/511)	0.04
**False-negative results in non-specific benign pathologies**[b]	15.3% (83/544)	10.1% (43/425)	0.02
**Pneumothorax**	21.8% (547/2505)	21.8 (471/2163)	0.99
**Pneumothorax requiring chest catheter drainage**	1.8% (45/2505)	2.4 (52/2163)	0.18
**Hemoptysis**	10.5% (264/2505)	10.7% (231/2163)	0.91

[a] From 1153 non-diagnostic pathologies with decidable reference standards.

[b] From 969 non-specific benign pathologies with decidable reference standards.

Table S3. Detailed Results of Percutaneous Transthoracic Needle Biopsy with False-Negative Results

	**Never or light smokers (n=128)**	**Moderate smokers (n=54)**	**Heavy smokers (n=54)**	***p*-value**
**Category of non-diagnostic pathologies**				<0.001
Non-specific benign pathologies (n=126)	65 (50.8%)	32 (59.3%)	29 (53.7%)	
Atypical cells (n=106)	62 (48.4%)	20 (37.0%)	24 (44.4%)	
Insufficient specimens (n=4)	1 (0.8%)	2 (3.7%)	1 (1.9%)	
**Category of final pathologies**				0.47
Adenocarcinoma (n=148)	102 (80.3%)	24 (44.4%)	22 (40.7%)	
Squamous cell carcinoma (n=55)	13 (10.2%)	19 (35.2%)	23 (42.6%)	
Small cell carcinoma (n=11)	6 (4.7%)	5 (9.3%)	0 (0.0%)	
Neuroendocrine neoplasm, other than small cell carcinoma (n=6)[a]	3 (2.4%)	1 (1.9%)	2 (3.7%)	
Lymphoma (n=5)	3 (2.4%)	1 (1.9%)	1 (1.9%)	
Others (n=11)[b]	1 (0.8%)	4 (7.4%)	6 (11.1%)	

[a] "Neuroendocrine neoplasm, other than small cell carcinoma" included typical or atypical carcinoid, or large cell neuroendocrine carcinoma.

[b] The "others" category included adenosquamous carcinoma or sarcomatoid carcinoma.

Table S4. Detailed Results of Logistic Regression Analysis After Adjusting for Covariates

	Adjusted OR	95% CI	*p*-value
**Diagnosis of malignancies (n=4668)**			
**Former smoker**	1.14	0.90 – 1.44	0.27
**Current smoker**	1.31	1.02 – 1.69	0.03
**Moderate smoker: Pack-year (> 20, ≤ 40)**	1.17	0.94 – 1.46	0.17
**Heavy smoker: Pack-year (> 40)**	1.22	0.96 – 1.56	0.11
**Older age**	1.03	1.03 – 1.04	<0.001
**Men**	0.60	0.49 – 0.72	<0.001
**Right middle lobe**	0.98	0.75 – 1.29	0.90
**Right lower lobe**	0.98	0.82 – 1.17	0.80
**Left upper lobe**	1.18	0.98 – 1.41	0.08
**Left lower lobe**	1.05	0.87 – 1.28	0.61
**Larger size**	1.17	1.13 – 1.21	<0.001
**Solid lesion**	0.63	0.46 – 0.86	0.004
**Presence of spiculation**	2.63	2.18 – 3.19	<0.001
**Presence of emphysema**	1.47	1.15 – 1.89	0.002
**Presence of emphysema along the needle tract**	0.65	0.46 – 0.92	0.02
**Presence of the open bronchus sign**	0.89	0.76 – 1.04	0.15
**Longer needle indwelling time**	0.94	0.93 – 0.96	<0.001
**Biopsy performed by a radiologist with sufficient experience**	1.02	0.90 – 1.16	0.77
**Non-diagnostic pathologies (n=4539)[a, b]**			
**Former smoker**	1.17	0.83 – 1.64	0.37
**Current smoker**	0.97	0.67 – 1.39	0.85
**Moderate smoker: Pack-year (> 20, ≤ 40)**	1.08	0.78 – 1.49	0.64
**Heavy smoker: Pack-year (> 40)**	1.69	1.19 – 2.40	0.003
**Age (> 65 years)**	1.11	0.91 – 1.35	0.31
**Men**	1.09	0.83 – 1.43	0.55
**Size (> 1 cm, ≤ 2 cm)**	1.11	0.70 – 1.76	0.66
**Size (> 2 cm, ≤ 3 cm)**	0.87	0.54 – 1.39	0.56
**Size (> 3 cm)**	1.06	0.66 – 1.67	0.82
**Solid lesion**	0.46	0.30 – 0.71	<0.001
**Fine needle aspiration only**	3.46	1.65 – 7.04	0.001
**Tissue sampling (≥ 3 times)**	0.88	0.72 – 1.07	0.20
**Prone position**	0.95	0.78 – 1.16	0.61
**Lateral position**	1.27	0.01 – 18.57	0.89
**Presence of hemoptysis**	1.52	1.13 – 2.03	0.005
**Final diagnosis of malignancy**	0.023	0.019 – 0.028	<0.001
**Presence of emphysema along the needle tract**	1.61	1.07 – 2.40	0.02
**Presence of the open bronchus sign**	1.37	1.08 – 1.74	0.009

**Longer needle indwelling time**	1.06	1.03 – 1.09	<0.001
**Biopsy performed by a radiologist with sufficient experience**	0.75	0.63 – 0.91	0.003
**False-negative results in non-diagnostic pathologies (n=1153)[c]**			
**Former smoker**	1.72	0.89 – 3.30	0.10
**Current smoker**	2.64	1.32 – 5.28	0.006
**Moderate smoker: Pack-year (> 20, ≤ 40)**	1.74	0.98 – 3.13	0.06
**Heavy smoker: Pack-year (> 40)**	2.12	1.17 – 3.92	0.02
**Age (> 65 years)**	2.05	1.44 – 2.94	<0.001
**Men**	0.32	0.18 – 0.55	<0.001
**Presence of emphysema**	1.86	1.03 – 3.31	0.04
**Upper or middle lobe**	0.72	0.51 – 1.00	0.05
**Solid lesion**	0.27	0.14 – 0.53	<0.001
**Non-diagnostic pathology with atypical cells**	11.38	7.68 – 17.06	<0.001
**Non-diagnostic pathology with insufficient specimens**	2.18	0.59 – 6.57	0.19
**Presence of emphysema along the needle tract**	0.96	0.44 – 2.09	0.92
**Presence of the open bronchus sign**	0.89	0.59 – 1.32	0.58
**Longer needle indwelling time**	1.03	0.99 – 1.06	0.10
**Biopsy performed by a radiologist with sufficient experience**	0.67	0.47 – 0.95	0.02
**False-negative results in non-specific benign pathologies (n=969)[d]**			
**Former smoker**	1.65	0.76 – 3.54	0.20
**Current smoker**	2.59	1.16 – 5.72	0.02
**Moderate smoker: Pack-year (> 20, ≤ 40)**	2.40	1.24 – 4.76	0.01
**Heavy smoker: Pack-year (> 40)**	2.38	1.19 – 4.90	0.02
**Age (> 65 years)**	2.27	1.49 – 3.48	<0.001
**Men**	0.34	0.17 – 0.66	0.002
**Presence of emphysema**	1.99	1.02 – 3.77	0.04
**Solid lesion**	0.24	0.11 – 0.54	<0.001
**Presence of emphysema along the needle tract**	0.69	0.28 – 1.63	0.40
**Presence of the open bronchus sign**	0.78	0.47 – 1.25	0.32
**Longer needle indwelling time**	1.02	0.98 – 1.06	0.34
**Biopsy performed by a radiologist with sufficient experience**	0.61	0.40 – 0.91	0.02
**Pneumothorax (n=4668)**			
**Former smoker**	1.01	0.77 – 1.33	0.92
**Current smoker**	0.96	0.71 – 1.28	0.77
**Moderate smoker: Pack-year (> 20, ≤ 40)**	1.24	0.96 – 1.59	0.10
**Heavy smoker: Pack-year (> 40)**	1.33	1.01 – 1.74	0.04
**Age (≥ 60 years)**	1.14	0.96 – 1.36	0.13
**Men**	1.07	0.86 – 1.34	0.53
**Right middle lobe**	1.76	1.31 – 2.36	<0.001

**Right lower lobe**	1.35	1.10 – 1.65	0.005
**Left upper lobe**	0.98	0.79 – 1.21	0.83
**Left lower lobe**	1.58	1.26 – 1.97	<0.001
**Size (> 2 cm)**	0.86	0.73 – 1.01	0.07
**Solid lesion**	0.92	0.66 – 1.32	0.65
**Presence of emphysema**	1.02	0.78 – 1.32	0.89
**Pleura-to-target (> 2 cm, ≤ 4 cm)**	1.40	1.19 – 1.66	<0.001
**Pleura-to-target (> 4 cm)**	1.89	1.53 – 2.32	<0.001
**Number of pleural passages (= 2)**	1.94	1.28 – 2.93	0.002
**Number of pleural passages (> 2)**	3.94	2.67 – 5.83	<0.001
**Presence of emphysema along the needle tract**	3.28	2.33 – 4.64	<0.001
**Presence of the open bronchus sign**	1.16	0.96 – 1.39	0.12
**Longer needle indwelling time**	1.06	1.04 – 1.08	<0.001
**Biopsy performed by a radiologist with sufficient experience**	1.07	0.92 – 1.23	0.38
**Pneumothorax requiring chest catheter drainage (n=4668)**			
**Former smoker**	1.49	0.72 – 2.98	0.27
**Current smoker**	1.66	0.77 – 3.43	0.18
**Moderate smoker: Pack-year (> 20, ≤ 40)**	0.92	0.45 – 1.92	0.81
**Heavy smoker: Pack-year (> 40)**	1.80	0.92 – 3.68	0.10
**Age (≥ 60 years)**	1.80	1.02 – 3.38	0.05
**Size (> 2 cm)**	0.80	0.51 – 1.28	0.34
**Solid lesion**	1.26	0.47 – 4.48	0.68
**Presence of emphysema**	1.02	0.47 – 2.04	0.96
**Pleura-to-target (> 2 cm, ≤ 4 cm)**	1.12	0.67 – 1.82	0.67
**Pleura-to-target (> 4 cm)**	1.45	0.83 – 2.47	0.18
**Number of pleural passages (= 2)**	1.76	0.69 – 4.01	0.21
**Number of pleural passages (> 2)**	5.77	2.56 – 11.76	<0.001
**Presence of emphysema along the needle tract**	4.38	2.07 – 9.96	<0.001
**Presence of the open bronchus sign**	1.46	0.86 – 2.39	0.15
**Longer needle indwelling time**	1.13	1.08 – 1.17	<0.001
**Biopsy performed by a radiologist with sufficient experience**	1.67	1.09 – 2.57	0.02
**Hemoptysis (n=4668)**			
**Former smoker**	0.88	0.61 – 1.26	0.48
**Current smoker**	0.64	0.42 – 0.96	0.04
**Moderate smoker: Pack-year (> 20, ≤ 40)**	0.81	0.55 – 1.19	0.28
**Heavy smoker: Pack-year (> 40)**	0.64	0.40 – 0.99	0.048
**Men**	0.62	0.46 – 0.82	<0.001
**Size (> 2 cm)**	0.68	0.56 – 0.84	<0.001
**Solid lesions**	0.48	0.35 – 0.67	<0.001
**Presence of emphysema**	1.06	0.68 – 1.60	0.79
**Pleura-to-target (> 2 cm, ≤ 4 cm)**	1.43	1.15 – 1.78	0.001

**Pleura-to-target (> 4 cm)**	2.21	1.70 – 2.87	<0.001
**Presence of emphysema along the needle tract**	0.78	0.39 – 1.49	0.46
**Presence of the open bronchus sign**	1.98	1.59 – 2.46	<0.001
**Longer needle indwelling time**	0.98	0.96 – 1.01	0.28
**Biopsy performed by a radiologist with sufficient experience**	1.07	0.88 – 1.30	0.48

Abbreviations: OR=odds ratio, CI=confidence interval.

[a] From 4539 PTNB procedures with decidable reference standards.

[b] The Firth correction was applied.

[c] From 1153 non-diagnostic pathologies with decidable reference standards.

[d] From 969 non-specific benign pathologies with decidable reference standards.

Table S5. Detailed Results of Logistic Regression Analysis After Adjusting for Covariates: Subgroup Analysis for Percutaneous Transthoracic Needle Biopsy with the Core Needle Biopsy Technique

	Adjusted OR	95% CI	*p*-value
**Diagnosis of malignancies (n=4608)**			
**Former smoker**	1.14	0.90 – 1.44	0.28
**Current smoker**	1.31	1.02 – 1.69	0.03
**Moderate smoker: Pack-year (> 20, ≤ 40)**	1.16	0.93 – 1.45	0.18
**Heavy smoker: Pack-year (> 40)**	1.20	0.94 – 1.53	0.15
**Older age**	1.03	1.03 – 1.04	<0.001
**Men**	0.61	0.50 – 0.74	<0.001
**Right middle lobe**	1.00	0.76 – 1.31	0.98
**Right lower lobe**	0.98	0.82 – 1.17	0.79
**Left upper lobe**	1.18	0.98 – 1.41	0.08
**Left lower lobe**	1.04	0.85 – 1.26	0.73
**Larger size**	1.17	1.12 – 1.21	<0.001
**Solid lesion**	0.65	0.47 – 0.88	0.006
**Presence of spiculation**	2.62	2.17 – 3.18	<0.001
**Presence of emphysema**	1.44	1.13 – 1.85	0.004
**Presence of emphysema along the needle tract**	0.64	0.46 – 0.91	0.01
**Presence of the open bronchus sign**	0.91	0.77 – 1.07	0.25
**Longer needle indwelling time**	0.94	0.93 – 0.96	<0.001
**Biopsy performed by a radiologist with sufficient experience**	1.02	0.90 – 1.16	0.78
**Non-diagnostic pathologies (n=4484)[a, b]**			
**Former smoker**	1.16	0.82 – 1.64	0.39
**Current smoker**	0.99	0.69 – 1.43	0.96
**Moderate smoker: Pack-year (> 20, ≤ 40)**	1.13	0.82 – 1.57	0.45
**Heavy smoker: Pack-year (> 40)**	1.75	1.23 – 2.50	0.002
**Age (> 65 years)**	1.10	0.90 – 1.34	0.37
**Men**	1.09	0.82 – 1.45	0.55
**Size (> 1 cm, ≤ 2 cm)**	1.08	0.67 – 1.71	0.76
**Size (> 2 cm, ≤ 3 cm)**	0.88	0.55 – 1.41	0.60
**Size (> 3 cm)**	1.08	0.67 – 1.72	0.76
**Solid lesion**	0.45	0.29 – 0.70	<0.001
**Tissue sampling (≥ 3 times)**	0.87	0.72 – 1.06	0.17
**Prone position**	0.93	0.76 – 1.13	0.48
**Lateral position**	1.22	0.01 – 18.11	0.90
**Presence of hemoptysis**	1.56	1.16 – 2.10	0.003
**Final diagnosis of malignancy**	0.022	0.018 – 0.027	<0.001

**Presence of emphysema along the needle tract**	1.62	1.07 – 2.42	0.02
**Presence of the open bronchus sign**	1.35	1.06 – 1.72	0.02
**Longer needle indwelling time**	1.06	1.03 – 1.09	<0.001
**Biopsy performed by a radiologist with sufficient experience**	0.75	0.62 – 0.91	0.003
**False-negative results in non-diagnostic pathologies (n=1126)[c]**			
**Former smoker**	1.78	0.90 – 3.50	0.10
**Current smoker**	2.70	1.31 – 5.52	0.007
**Moderate smoker: Pack-year (> 20, ≤ 40)**	1.86	1.04 – 3.38	0.04
**Heavy smoker: Pack-year (> 40)**	2.30	1.26 – 4.28	0.008
**Age (> 65 years)**	2.03	1.42 – 2.92	<0.001
**Men**	0.31	0.17 – 0.55	<0.001
**Presence of emphysema**	1.88	1.04 – 3.34	0.03
**Upper or middle lobe**	0.71	0.51 – 1.01	0.06
**Solid lesion**	0.24	0.12 – 0.48	<0.001
**Non-diagnostic pathology with atypical cells**	10.36	6.94 – 15.62	<0.001
**Non-diagnostic pathology with insufficient specimens**	1.95	0.41 – 6.95	0.34
**Presence of emphysema along the needle tract**	0.96	0.44 – 2.07	0.91
**Presence of the open bronchus sign**	0.81	0.53 – 1.22	0.31
**Longer needle indwelling time**	1.02	0.99 – 1.06	0.23
**Biopsy performed by a radiologist with sufficient experience**	0.67	0.47 – 0.95	0.02
**False-negative results in non-specific benign pathologies (n=956)[d]**			
**Former smoker**	1.52	0.69 – 3.31	0.29
**Current smoker**	2.36	1.04 – 5.30	0.04
**Moderate smoker: Pack-year (> 20, ≤ 40)**	2.48	1.28 – 4.98	0.008
**Heavy smoker: Pack-year (> 40)**	2.45	1.21 – 5.07	0.01
**Age (> 65 years)**	2.33	1.52 – 3.60	<0.001
**Men**	0.39	0.19 – 0.75	0.007
**Presence of emphysema**	1.98	1.01 – 3.76	0.04
**Solid lesion**	0.22	0.10 – 0.50	<0.001
**Presence of emphysema along the needle tract**	0.68	0.28 – 1.62	0.39
**Presence of the open bronchus sign**	0.72	0.42 – 1.17	0.20
**Longer needle indwelling time**	1.02	0.97 – 1.06	0.40
**Biopsy performed by a radiologist with sufficient experience**	0.60	0.40 – 0.91	0.02
**Pneumothorax (n=4608)**			
**Former smoker**	1.01	0.76 – 1.33	0.96
**Current smoker**	0.95	0.71 – 1.28	0.74
**Moderate smoker: Pack-year (> 20, ≤ 40)**	1.24	0.97 – 1.60	0.09
**Heavy smoker: Pack-year (> 40)**	1.33	1.01 – 1.74	0.04

**Age (≥ 60 years)**	1.14	0.96 – 1.36	0.13
**Men**	1.10	0.87 – 1.37	0.43
**Right middle lobe**	1.68	1.25 – 2.26	<0.001
**Right lower lobe**	1.30	1.06 – 1.60	0.01
**Left upper lobe**	0.97	0.78 – 1.20	0.76
**Left lower lobe**	1.51	1.20 – 1.88	<0.001
**Size (> 2 cm)**	0.86	0.73 – 1.02	0.07
**Solid lesion**	0.82	0.65 – 1.32	0.64
**Presence of emphysema**	1.01	0.78 – 1.31	0.91
**Pleura-to-target (> 2 cm, ≤ 4 cm)**	1.41	1.19 – 1.66	<0.001
**Pleura-to-target (> 4 cm)**	1.92	1.56 – 2.36	<0.001
**Number of pleural passages (= 2)**	1.94	1.26 – 2.98	0.002
**Number of pleural passages (> 2)**	4.01	2.71 – 5.93	<0.001
**Presence of emphysema along the needle tract**	3.29	2.33 – 4.65	<0.001
**Presence of the open bronchus sign**	1.16	0.97 – 1.40	0.11
**Longer needle indwelling time**	1.07	1.04 – 1.09	<0.001
**Biopsy performed by a radiologist with sufficient experience**	1.07	0.92 – 1.24	0.36
**Pneumothorax requiring chest catheter drainage (n=4608)**			
**Former smoker**	1.54	0.74 – 3.09	0.23
**Current smoker**	1.73	0.80 – 3.58	0.15
**Moderate smoker: Pack-year (> 20, ≤ 40)**	0.92	0.45 – 1.92	0.81
**Heavy smoker: Pack-year (> 40)**	1.78	0.91 – 3.64	0.10
**Age (≥ 60 years)**	1.79	1.01 – 3.37	0.06
**Size (> 2 cm)**	0.77	0.49 – 1.24	0.28
**Solid lesion**	1.30	0.48 – 4.62	0.65
**Presence of emphysema**	1.01	0.46 – 2.03	0.97
**Pleura-to-target (> 2 cm, ≤ 4 cm)**	1.16	0.70 – 1.90	0.55
**Pleura-to-target (> 4 cm)**	1.52	0.87 – 2.59	0.13
**Number of pleural passages (= 2)**	1.77	0.69 – 4.09	0.21
**Number of pleural passages (> 2)**	5.80	2.57 – 11.86	<0.001
**Presence of emphysema along the needle tract**	4.37	2.06 – 9.93	<0.001
**Presence of the open bronchus sign**	1.53	0.90 – 2.50	0.10
**Longer needle indwelling time**	1.13	1.08 – 1.17	<0.001
**Biopsy performed by a radiologist with sufficient experience**	1.71	1.11 – 2.64	0.02
**Hemoptysis (n=4608)**			
**Former smoker**	0.90	0.62 – 1.29	0.56
**Current smoker**	0.66	0.43 – 1.00	0.05
**Moderate smoker: Pack-year (> 20, ≤ 40)**	0.83	0.56 – 1.22	0.33
**Heavy smoker: Pack-year (> 40)**	0.65	0.41 – 1.01	0.06
**Men**	0.60	0.45 – 0.79	<0.001
**Size (> 2 cm)**	0.67	0.55 – 0.83	<0.001

**Solid lesions**	0.48	0.34 – 0.67	<0.001
**Presence of emphysema**	1.05	0.67 – 1.59	0.81
**Pleura-to-target (> 2 cm, ≤ 4 cm)**	1.43	1.15 – 1.78	0.001
**Pleura-to-target (> 4 cm)**	2.25	1.72 – 2.92	<0.001
**Presence of emphysema along the needle tract**	0.78	0.39 – 1.49	0.46
**Presence of the open bronchus sign**	2.00	1.60 – 2.49	<0.001
**Longer needle indwelling time**	0.99	0.96 – 1.01	0.36
**Biopsy performed by a radiologist with sufficient experience**	1.08	0.89 – 1.31	0.43

Abbreviations: OR=odds ratio, CI=confidence interval.

[a] From 4539 PTNB procedures with decidable reference standards.

[b] The Firth correction was applied.

[c] From 1153 non-diagnostic pathologies with decidable reference standards.

[d] From 969 non-specific benign pathologies with decidable reference standards.

Table S6. Results of Multivariable Logistic Regression with Backward Elimination in All Patients

	Adjusted OR	95% CI	*p*-value
**Diagnosis of malignancies (n=4668)**			
**Moderate smoker: Pack-year (> 20, ≤ 40)**	1.40	1.15 – 1.69	<0.001
**Heavy smoker: Pack-year (> 40)**	1.52	1.22 – 1.89	<0.001
**Age (> 65 years)**	1.70	1.49 – 1.94	<0.001
**Men**	0.65	0.55 – 0.76	<0.001
**Size (> 2 cm)**	2.01	1.75 – 2.31	<0.001
**Solid lesion**	0.63	0.46 – 0.86	0.004
**Presence of emphysema**	1.58	1.25 – 2.03	<0.001
**Presence of spiculation**	2.43	2.01 – 2.93	<0.001
**Presence of emphysema along the needle tract**	0.61	0.43 – 0.87	0.005
**Presence of the open bronchus sign**	0.86	0.73 – 1.01	0.06
**Longer needle indwelling time**	0.94	0.93 – 0.96	<0.001
**Non-diagnostic pathologies (n=4539)[a]**			
**Moderate smoker: Pack-year (> 20, ≤ 40)**	1.14	0.88 – 1.48	0.31
**Heavy smoker: Pack-year (> 40)**	1.89	1.40 – 2.54	<0.001
**Solid lesion**	0.52	0.35 – 0.81	0.003
**Presence of emphysema**	1.41	1.06 – 1.88	0.02
**18G needle**	0.40	0.32 – 0.49	<0.001
**Fine needle aspiration only**	3.72	1.78 – 7.58	<0.001
**Presence of hemoptysis**	1.28	0.95 – 1.72	0.10
**Final diagnosis of malignancy**	0.021	0.017 – 0.025	<0.001
**Presence of the open bronchus sign**	1.34	1.06 – 1.70	0.02
**Longer needle indwelling time**	1.07	1.05 – 1.10	<0.001
**Biopsy performed by a radiologist with sufficient experience**	0.74	0.61 – 0.89	0.002
**False-negative results in non-diagnostic pathologies (n=1153)[b]**			
**Former smoker**	1.74	0.90 – 3.35	0.10
**Current smoker**	2.71	1.35 – 5.42	0.005
**Moderate smoker: Pack-year (> 20, ≤ 40)**	1.81	1.01 – 3.26	0.047
**Heavy smoker: Pack-year (> 40)**	2.27	1.25 – 4.19	0.008
**Age (> 65 years)**	2.01	1.41 – 2.88	<0.001
**Men**	0.30	0.17 – 0.52	<0.001
**Upper or middle lobe**	0.72	0.51 – 1.01	0.06
**Solid lesion**	0.24	0.13 – 0.47	<0.001
**Presence of emphysema**	1.80	1.11 – 2.90	0.02
**18G needle**	1.39	0.94 – 2.04	0.10
**Fine needle aspiration only**	4.05	1.55 – 10.53	0.004
**Presence of pneumothorax**	1.48	1.01 – 2.16	0.04

**Non-diagnostic pathology with atypical cells**	11.38	7.64 – 17.13	<0.001
**Non-diagnostic pathology with insufficient specimens**	1.48	0.38 – 4.67	0.53
**Biopsy performed by a radiologist with sufficient experience**	0.67	0.47 – 0.94	0.02
**False-negative results in non-specific benign pathologies (n=969)[c]**			
**Former smoker**	1.55	0.71 – 3.31	0.27
**Current smoker**	2.63	1.19 – 5.80	0.02
**Moderate smoker: Pack-year (> 20, ≤ 40)**	2.35	1.22 – 4.69	0.01
**Heavy smoker: Pack-year (> 40)**	2.32	1.15 – 4.80	0.02
**Age (> 65 years)**	2.19	1.43 – 3.37	<0.001
**Men**	0.33	0.16 – 0.63	0.001
**Upper or middle lobe**	0.70	0.47 – 1.05	0.09
**Solid lesion**	0.24	0.12 – 0.52	<0.001
**Presence of emphysema**	1.72	0.98 – 2.97	0.05
**18G needle**	1.55	1.001 – 2.39	0.047
**Presence of pneumothorax**	1.70	1.08 – 2.62	0.02
**Biopsy performed by a radiologist with sufficient experience**	0.62	0.41 – 0.92	0.02
**Pneumothorax (n=4668)**			
**Moderate smoker: Pack-year (> 20, ≤ 40)**	1.24	1.03 – 1.49	0.02
**Heavy smoker: Pack-year (> 40)**	1.28	1.04 – 1.57	0.02
**Age (> 65 years)**	1.25	1.08 – 1.45	0.004
**Upper or middle lobe**	0.76	0.65 – 0.88	<0.001
**Size (> 2 cm)**	0.85	0.72 – 1.00	0.0497
**Pleura-to-target (> 2 cm, ≤ 4 cm)**	1.45	1.22 – 1.71	<0.001
**Pleura-to-target (> 4 cm)**	2.01	1.63 – 2.47	<0.001
**18G needle**	0.79	0.67 – 0.92	0.003
**Multiple pleural passage**	2.67	2.00 – 3.56	<0.001
**Presence of hemoptysis**	0.56	0.42 – 0.73	<0.001
**Presence of emphysema along the needle tract**	3.07	2.32 – 4.05	<0.001
**Presence of the open bronchus sign**	1.17	0.98 – 1.40	0.08
**Longer needle indwelling time**	1.06	1.04 – 1.08	<0.001
**Pneumothorax requiring chest catheter drainage (n=4668)[d]**			
**Moderate smoker: Pack-year (> 20, ≤ 40)**	0.95	0.51 – 1.75	0.86
**Heavy smoker: Pack-year (> 40)**	1.73	0.97 – 3.12	0.07
**Age (> 65 years)**	1.54	0.98 – 2.48	0.06
**Men**	1.90	1.04 – 3.52	0.04
**Prone position**	0.62	0.41 – 0.95	0.03
**Lateral position**	14.38	0.10 – 186.38	0.20
**Multiple pleural passage**	3.40	1.78 – 6.16	<0.001
**Presence of emphysema along the needle tract**	4.51	2.61 – 7.61	<0.001
**Longer needle indwelling time**	1.12	1.08 – 1.16	<0.001

**Biopsy performed by a radiologist with sufficient experience**	1.58	1.04 – 2.41	0.03
**Hemoptysis (n=4668)[d]**			
**Former smoker**	0.76	0.55 – 1.06	0.11
**Current smoker**	0.56	0.39 – 0.80	0.002
**Men**	0.62	0.47 – 0.83	0.001
**Size (> 2 cm)**	0.73	0.60 – 0.91	0.004
**Solid lesion**	0.61	0.44 – 0.87	0.006
**Pleura-to-target (> 2 cm, ≤ 4 cm)**	1.42	1.13 – 1.77	0.002
**Pleura-to-target (> 4 cm)**	2.42	1.84 – 3.18	<0.001
**Prone position**	1.11	0.91 – 1.37	0.31
**Lateral position**	33.38	2.35 – 4778.64	0.009
**18G needle**	0.33	0.26 – 0.42	<0.001
**Multiple pleural passage**	0.65	0.40 – 1.01	0.05
**Tissue sampling (≥ 3 times)**	0.63	0.52 – 0.78	<0.001
**Presence of pneumothorax**	0.55	0.42 – 0.72	<0.001
**Presence of the open bronchus sign**	1.83	1.46 – 2.28	<0.001

Abbreviations: OR=odds ratio, CI=confidence interval.

[a] From 4539 PTNB procedures with decidable reference standards.

[b] From 1153 non-diagnostic pathologies with decidable reference standards.

[c] From 969 non-specific benign pathologies with decidable reference standards.

[d] The Firth correction was applied.

Table S7. Results of Multivariable Logistic Regression with Backward Elimination Regarding the Effect of Lung Cancer Screening Eligibility on Percutaneous Transthoracic Needle Biopsy

	USPSTF			NCCN			NLST		
	**Adjusted OR**	**95% CI**	***P*-value**	**Adjusted OR**	**95% CI**	***P*-value**	**Adjusted OR**	**95% CI**	***p*-value**
**Diagnosis of malignancies (n=4668)**									
**Screening eligibility**	1.39	1.18 – 1.64	<0.001	1.56	1.32 – 1.85	<0.001	1.31	1.09 – 1.58	0.004
**Men**	0.70	0.61 – 0.82	<0.001	0.64	0.54 – 0.75	<0.001	0.75	0.65 – 0.86	<0.001
**Size (> 2 cm)**	2.07	1.80 – 2.37	<0.001	2.05	1.79 – 2.35	<0.001	2.07	1.81 – 2.38	<0.001
**Solid lesion**	0.64	0.46 – 0.86	0.004	0.63	0.46 – 0.86	0.004	0.63	0.46 – 0.86	0.004
**Presence of emphysema**	1.79	1.41 – 2.28	<0.001	1.72	1.36 – 2.20	<0.001	1.85	1.46 – 2.35	<0.001
**Presence of spiculation**	2.40	2.00 – 2.91	<0.001	2.41	2.00 – 2.91	<0.001	2.42	2.01 – 2.92	<0.001
**Presence of emphysema along the needle tract**	0.63	0.44 – 0.89	0.009	0.62	0.44 – 0.88	0.008	0.64	0.46 – 0.91	0.01
**Presence of the open bronchus sign**	0.87	0.74 – 1.02	0.08	0.87	0.74 – 1.02	0.09	0.86	0.74 – 1.01	0.07
**Longer needle indwelling time**	0.95	0.93 – 0.96	<0.001	0.95	0.93 – 0.96	<0.001	0.95	0.93 – 0.96	<0.001
**Non-diagnostic pathologies (n=4539)[a]**									
**Screening eligibility**	N/I			1.32	1.07 – 1.65	0.01	N/I		
**Men**	1.31	1.06 – 1.60	0.01	N/I			1.31	1.06 – 1.60	0.01
**Solid lesion**	0.55	0.36 – 0.84	0.005	0.53	0.35 – 0.81	0.003	0.55	0.36 – 0.84	0.005
**Presence of emphysema**	1.54	1.17 – 2.03	0.002	1.48	1.12 – 1.97	0.006	1.54	1.17 – 2.03	0.002
**18G needle**	0.40	0.32 – 0.49	<0.001	0.39	0.32 – 0.48	<0.001	0.40	0.32 – 0.49	<0.001
**Fine needle aspiration only**	3.74	1.78 – 7.63	<0.001	3.64	1.75 – 7.40	<0.001	3.74	1.78 – 7.63	<0.001
**Presence of hemoptysis**	1.29	0.96 – 1.73	0.09	N/I			1.29	0.96 – 1.73	0.09
**Final diagnosis of malignancy**	0.022	0.018 – 0.027	<0.001	0.021	0.017 – 0.026	<0.001	0.022	0.018 – 0.027	<0.001
**Presence of the open bronchus sign**	1.32	1.04 – 1.68	0.02	1.35	1.06 – 1.71	0.01	1.32	1.04 – 1.68	0.02
**Longer needle indwelling time**	1.07	1.05 – 1.10	<0.001	1.07	1.05 – 1.10	<0.001	1.07	1.05 – 1.10	<0.001

**Biopsy performed by a radiologist with sufficient experience**	0.74	0.61 – 0.90	0.002	0.74	0.62 – 0.90	0.002	0.74	0.61 – 0.90	0.002
**False-negative results in non-diagnostic pathologies (n=1153)[b]**									
**Screening eligibility**	1.79	1.16 – 2.76	0.008	2.17	1.38 – 3.45	<0.001	1.53	0.96 – 2.41	0.07
**Men**	0.62	0.41 – 0.92	0.02	0.52	0.33 – 0.81	0.004	0.70	0.48 – 1.03	0.07
**Upper or middle lobe**	0.71	0.51 – 0.99	0.04	0.71	0.51 – 0.99	0.04	0.71	0.51 – 0.997	0.048
**Solid lesion**	0.29	0.15 – 0.54	<0.001	0.28	0.15 – 0.54	<0.001	0.29	0.16 – 0.55	<0.001
**Presence of emphysema**	2.50	1.57 – 3.96	<0.001	2.35	1.47 – 3.75	<0.001	2.60	1.63 – 4.13	<0.001
**Fine needle aspiration only**	3.69	1.42 – 9.35	0.006	3.91	1.51 – 9.92	0.004	3.64	1.40 – 9.29	0.007
**Presence of pneumothorax**	1.44	0.99 – 2.09	0.05	1.44	0.98 – 2.08	0.06	1.46	1.002 – 2.11	0.047
**Non-diagnostic pathology with atypical cells**	10.65	7.26 – 15.77	<0.001	10.63	7.24 – 15.77	<0.001	10.71	7.31 – 15.86	<0.001
**Non-diagnostic pathology with insufficient specimens**	1.47	0.38 – 4.55	0.53	1.50	0.38 – 4.67	0.51	1.44	0.37 – 4.45	0.555
**Biopsy performed by a radiologist with sufficient experience**	0.66	0.47 – 0.92	0.02	0.67	0.48 – 0.94	0.02	0.66	0.47 – 0.93	0.017
**False-negative results in non-specific benign pathologies (n=969)[c]**									
**Screening eligibility**	2.17	1.31 – 3.63	0.003	2.50	1.48 – 4.35	<0.001	1.79	1.09 – 2.89	0.02
**Men**	0.62	0.38 – 1.01	0.06	0.52	0.30 – 0.89	0.02	N/I		
**Upper or middle lobe**	0.70	0.47 – 1.04	0.08	0.70	0.47 – 1.04	0.08	0.70	0.48 – 1.04	0.08
**Solid lesion**	0.26	0.13 – 0.57	<0.001	0.26	0.13 – 0.56	<0.001	0.27	0.13 – 0.57	<0.001
**Presence of emphysema**	2.43	1.41 – 4.14	0.001	2.37	1.38 – 4.03	0.002	2.30	1.36 – 3.82	0.001
**18G needle**	1.55	1.01 – 2.36	0.04	1.55	1.01 – 2.37	0.04	1.52	0.99 – 2.30	0.05
**Tissue sampling (≥ 3 times)**	N/I			N/I			0.71	0.48 – 1.04	0.08
**Presence of pneumothorax**	1.72	1.11 – 2.64	0.01	1.72	1.11 – 2.63	0.01	1.75	1.13 – 2.68	0.01
**Biopsy performed by a radiologist with sufficient experience**	0.60	0.40 – 0.89	0.01	0.62	0.41 – 0.92	0.02	0.59	0.39 – 0.88	0.01
**Pneumothorax (n=4668)**									
**Screening eligibility**	1.23	1.05 – 1.45	0.01	1.30	1.11 – 1.51	0.001	N/I		
**Men**	N/I			N/I			1.17	1.01 – 1.37	0.04
**Upper or middle lobe**	0.75	0.65 – 0.87	<0.001	0.75	0.65 – 0.87	<0.001	0.76	0.65 – 0.88	<0.001

**Size (> 2 cm)**	0.87	0.74 – 1.02	0.09	0.86	0.73 – 1.01	0.07	0.87	0.74 – 1.02	0.09
**Pleura-to-target (> 2 cm, ≤ 4 cm)**	1.44	1.22 – 1.70	<0.001	1.44	1.22 – 1.70	<0.001	1.44	1.22 – 1.70	<0.001
**Pleura-to-target (> 4 cm)**	2.02	1.64 – 2.48	<0.001	2.01	1.63 – 2.47	<0.001	2.01	1.64 – 2.47	<0.001
**18G needle**	0.79	0.67 – 0.92	0.003	0.79	0.67 – 0.92	0.003	0.79	0.67 – 0.92	0.003
**Multiple pleural passage**	2.65	1.98 – 3.54	<0.001	2.67	2.00 – 3.56	<0.001	2.65	1.98 – 3.54	<0.001
**Presence of hemoptysis**	0.55	0.42 – 0.72	<0.001	0.56	0.42 – 0.73	<0.001	0.56	0.42 – 0.72	<0.001
**Presence of emphysema along the needle tract**	3.29	2.50 – 4.33	<0.001	3.18	2.41 – 4.19	<0.001	3.39	2.58 – 4.45	<0.001
**Presence of the open bronchus sign**	1.17	0.98 – 1.40	0.08	1.18	0.98 – 1.40	0.07	1.17	0.98 – 1.39	0.09
**Longer needle indwelling time**	1.06	1.04 – 1.08	<0.001	1.06	1.04 – 1.08	<0.001	1.06	1.04 – 1.08	<0.001
**Pneumothorax requiring chest catheter drainage (n=4668)[c]**									
**Screening eligibility**	N/I			N/I			N/I		
**Men**	2.28	1.38 – 3.93	0.001	2.28	1.38 – 3.93	0.001	2.28	1.38 – 3.93	0.001
**Prone position**	0.61	0.40 – 0.93	0.02	0.61	0.40 – 0.93	0.02	0.61	0.40 – 0.93	0.02
**Lateral position**	13.95	0.10 – 182.67	0.20	13.95	0.10 – 182.67	0.20	13.95	0.10 – 182.67	0.20
**Multiple pleural passage**	3.24	1.70 – 5.86	<0.001	3.24	1.70 – 5.86	<0.001	3.24	1.70 – 5.86	<0.001
**Presence of emphysema along the needle tract**	5.39	3.20 – 8.86	<0.001	5.39	3.20 – 8.86	<0.001	5.39	3.20 – 8.86	<0.001
**Longer needle indwelling time**	1.11	1.08 – 1.15	<0.001	1.11	1.08 – 1.15	<0.001	1.11	1.08 – 1.15	<0.001
**Biopsy performed by a radiologist with sufficient experience**	1.56	1.03 – 2.38	0.04	1.56	1.03 – 2.38	0.04	1.56	1.03 – 2.38	0.04
**Hemoptysis (n=4668)[c]**									
**Screening eligibility**	0.78	0.58 – 1.05	0.10	0.72	0.54 – 0.97	0.03	0.68	0.47 – 0.96	0.03
**Men**	0.52	0.41 – 0.65	<0.001	0.56	0.43 – 0.71	<0.001	0.52	0.42 – 0.64	<0.001
**Size (> 2 cm)**	0.73	0.60 – 0.90	0.004	0.74	0.60 – 0.91	0.004	0.73	0.60 – 0.90	0.004
**Solid**	0.61	0.43 – 0.86	0.005	0.61	0.43 – 0.86	0.005	0.61	0.44 – 0.86	0.006
**Pleura-to-target (> 2 cm, ≤ 4 cm)**	1.43	1.14 – 1.79	0.002	1.44	1.15 – 1.79	0.002	1.43	1.14 – 1.78	0.002
**Pleura-to-target (> 4 cm)**	2.41	1.83 – 3.16	<0.001	2.41	1.83 – 3.16	<0.001	2.41	1.83 – 3.16	<0.001
**Prone position**	1.11	0.90 – 1.36	0.34	1.10	0.90 – 1.36	0.35	1.11	0.90 – 1.36	0.34
**Lateral position**	33.95	2.39 – 4862.47	0.009	33.63	2.37 – 4814.85	0.009	34.14	2.40 – 4888.76	0.008

**18G needle**	0.33	0.25 – 0.42	<0.001	0.33	0.25 – 0.42	<0.001	0.33	0.25 – 0.42	<0.001
**Multiple pleural passage**	0.66	0.41 – 1.02	0.06	0.65	0.40 – 1.01	0.06	0.66	0.41 – 1.03	0.07
**Tissue sampling (≥ 3 times)**	0.63	0.52 – 0.77	<0.001	0.63	0.52 – 0.77	<0.001	0.63	0.52 – 0.77	<0.001
**Presence of pneumothorax**	0.55	0.42 – 0.72	<0.001	0.56	0.42 – 0.72	<0.001	0.55	0.42 – 0.72	<0.001
**Presence of the open bronchus sign**	1.83	1.46 – 2.28	<0.001	1.83	1.46 – 2.28	<0.001	1.83	1.46 – 2.27	<0.001

Abbreviations: USPSTF=U.S. Preventive Service Task Force, NCCN= National Comprehensive Cancer Network, NLST=National Lung Screening Trial, OR=odds ratio, CI=confidence interval; N/I=not included in final logistic regression model.

[a] From 4539 PTNB procedures with decidable reference standards.

[b] From 1153 non-diagnostic pathologies with decidable reference standards.

[c] The Firth correction was applied.
